# SigB modulates expression of novel SigB regulon members via Bc1009 in non-stressed and heat-stressed cells revealing its alternative roles in *Bacillus cereus*

**DOI:** 10.1186/s12866-023-02783-3

**Published:** 2023-02-10

**Authors:** Kah Yen Claire Yeak, Marcel Tempelaars, Jia Lun Wu, Wouter Westerveld, Alexander Reder, Stephan Michalik, Vishnu M. Dhople, Uwe Völker, Jan Pané-Farré, Marjon H. J. Wells-Bennik, Tjakko Abee

**Affiliations:** 1 NIZO, Kernhemseweg 2, PO Box 20, 6718 ZB Ede, The Netherlands; 2 Food Microbiology, Wageningen University and Research, PO Box 8129, 6700 EV Wageningen, The Netherlands; 3 Interfaculty Institute for Genetics and Functional Genomics, University Medicine Greifswald, Greifswald, Germany; 4 Center for Synthetic Microbiology (SYNMIKRO) & Department of Chemistry, Philipps-University Marburg, Karl-Von-Frisch-Strasse 14, 35043 Marburg, Germany

**Keywords:** Adaptive general stress response, Phosphocarrier protein, Motility, Sporulation, Virulence, Metabolic crosstalk, SigB baseline function, Amino acid metabolisms, SigB subregulon

## Abstract

**Background:**

The *Bacillus cereus* Sigma B (SigB) dependent general stress response is activated via the two-component RsbKY system, which involves a phosphate transfer from RsbK to RsbY. It has been hypothesized that the Hpr-like phosphocarrier protein (Bc1009) encoded by *bc1009* in the SigB gene cluster may play a role in this transfer, thereby acting as a regulator of SigB activation. Alternatively, Bc1009 may be involved in the activation of a subset of SigB regulon members.

**Results:**

We first investigated the potential role of *bc1009* to act as a SigB regulator but ruled out this possibility as the deletion of *bc1009* did not affect the expression of *sigB* and other SigB gene cluster members. The SigB-dependent functions of Bc1009 were further examined in *B. cereus* ATCC14579 via comparative proteome profiling (backed up by transcriptomics) of wt, Δ*bc1009* and Δ*sigB* deletion mutants under heat stress at 42 °C. This revealed 284 proteins displaying SigB-dependent alterations in protein expression levels in heat-stressed cells, including a subgroup of 138 proteins for which alterations were also Bc1009-dependent. Next to proteins with roles in stress defense, newly identified SigB and Bc1009-dependent proteins have roles in cell motility, signal transduction, transcription, cell wall biogenesis, and amino acid transport and metabolism. Analysis of lethal stress survival at 50 °C after pre-adaptation at 42 °C showed intermediate survival efficacy of *Δbc1009* cells, highest survival of wt, and lowest survival of *ΔsigB* cells, respectively. Additional comparative proteome analysis of non-stressed wt and mutant cells at 30 °C revealed 96 proteins with SigB and Bc1009-dependent differences in levels: 51 were also identified under heat stress, and 45 showed significant differential expression at 30 °C. This includes proteins with roles in carbohydrate/ion transport and metabolism. Overlapping functions at 30 °C and 42 °C included proteins involved in motility, and Δ*sigB* and Δ*bc1009* cells showed reduced motility compared to wt cells in swimming assays at both temperatures.

**Conclusion:**

Our results extend the *B. cereus* SigB regulon to > 300 members, with a novel role of SigB-dependent Bc1009 in the activation of a subregulon of  > 180 members, conceivably via interactions with other transcriptional regulatory networks.

**Supplementary Information:**

The online version contains supplementary material available at 10.1186/s12866-023-02783-3.

## Background

*Bacillus cereus* is a Gram-positive endospore-forming facultative anaerobic bacterium found in soil, invertebrates, plants, and in fresh and stored foods [1]. It is a foodborne pathogen that can cause emetic and diarrheal disease due to the production of the emetic toxin cereulide in foods [1, 2], or the production of diarrhoeal toxins in the human intestine once foods contaminated with *B. cereus* are ingested. The diarrhoeal symptoms can involve non-hemolytic and hemolytic enterotoxin (Nhe and Hbl, respectively), and cytotoxin K [1, 3, 4].

Environmental transmission of *B. cereus* is strongly supported by the production of highly stress-resistant spores [1], while the resistance of vegetative cells to various stress conditions is enhanced by the activation of the so-called general stress response (GSR) [5]. This includes stresses that are encountered during food processing and preservation [6, 7]. In Gram-positive bacteria, including *B. cereus*, the GSR is governed by the master regulator of stress, i.e., the alternative sigma factor B (SigB) [5, 8]. Upon exposure to environmental stresses and nutrient limitation, SigB is triggered to reprogram the transcriptional and translational machinery in the cells, resulting in the production of proteins that mediate increased survival [8–11].

In *B. cereus,* SigB is activated via a two-component system, comprising the signal-receiving sensor kinase RsbK and its cognate response regulator, RsbY phosphatase [12, 13]. In the presence of stressors, RsbK autophosphorylates its histidine residue and initiates the transfer of a phosphate group to the C-terminal receiver (REC) domain of the RsbY protein [12, 14]. The phosphorylated RsbY then dephosphorylates the anti-sigma factor antagonist RsbV, increasing its affinity to the anti-sigma factor RsbW and their subsequent association. The formation of RsbVW complexes releases RsbW from SigB, leading to SigB activation [12, 14, 15]. Without stressors, the methyltransferase RsbM methylates the S-helix of RsbK and prohibits its phosphate transfer to RsbY [14]. The unphosphorylated RsbY does not dephosphorylate RsbV; thus, RsbW remains bound to SigB to keep SigB inactive.

In *B. subtilis*, SigB activity is controlled by two pathways, which independently sense energy and environmental stresses. Both pathways converge to the anti-anti-sigma factor RsbV [16–19]. Sensing energy stress, such as low adenosine triphosphate (ATP) levels, requires the activity of the hydrolase RsbQ and phosphatase RsbP, although the specific signal is unknown [18, 20, 21]. Environmental stress uses the stressosome complex consisting of the RsbR, RsbS, and RsbT sensor proteins. Once environmental stress is sensed, RsbT is activated and released from the stressosome, activating the phosphatase RsbU. Active RsbU dephosphorylates RsbV, in analogy to *B. cereus* RsbY, promoting the partner switching of RsbW bound to SigB to the anti-anti-sigma factor RsbV, resulting in SigB activation [8, 11, 22].

*B. cereus* uses the RsbKY system to sense environmental and nutritional cues [5]*.* Temperature upshift induces the strongest SigB GSR in *B. cereus*. Ethanol exposure and osmotic upshock activate SigB moderately, while energy stress (i.e., ATP depletion) activates SigB only mildly [5]*.* Upon SigB activation, around 30 genes have been reported to be upregulated by SigB in *B. cereus* [5, 10, 12], and these genes are referred to as the SigB regulon members. The reported RsbKY-controlled SigB regulon in *B. cereus* and its group members [23] is relatively small in comparison to the stressosome-controlled SigB regulons in *B. subtilis* [8, 24] and *Listeria monocytogenes* [25, 26], which both have at least > 300 SigB regulon members described to date. Alternative functions of SigB other than its role in the GSR have also been reported for *B. subtilis,* i.e., biofilm formation, sporulation, and fungal control [27–30], and for *L. monocytogenes*, i.e., virulence, antibiotic resistance and carbon metabolism [26, 31, 32].

It has previously been suggested that a Hpr-like phosphocarrier protein encoded by *bc1009*, located in the SigB gene cluster, may affect the efficacy of the RsbKY stress sensor and SigB activation [12]. However, so far, no evidence has been provided to substantiate this suggestion, and alternative roles of *B. cereus* SigB remain to be identified. Therefore, in this study, we used comparative proteome profiling, backed up by transcriptomics, to determine the *B. cereus* SigB regulon following quantification of differentially expressed genes/proteins in *B. cereus* ATCC14579 wt versus *sigB* (Δ*sigB*) and *bc1009* mutants (Δ*bc1009*) in non-heat-stressed and heat-stressed cells at 30 °C and 42 °C, respectively. The approach combined with selected phenotyping experiments provides further insight into the *B. cereus* SigB regulon composition and the possible roles of Bc1009 in RsbKY-induced SigB activation and the control of SigB regulon members.

## Materials and methods

### Strains, media, and growth conditions

*Bacillus cereus* ATCC14579 wild-type (wt) strain (laboratory stock) and its isogenic mutant strains Δ*sigB* and Δ*bc1009* were used in this study (Table 1). The ATCC14579 strain contains virulence genes encoding Nhe, Hbl and CytK, but not the *ces* gene cluster for cereulide production. The two mutants were constructed as described by Warda et al. [6]. Bacteria were routinely cultured in Brain Heart Infusion (BHI) medium (Becton, Dickinson Difco, Breda, The Netherlands) with incubation at 30 °C and 200 rpm, unless otherwise stated. Overnight (ON) cultures were prepared by inoculating strains from -80ºC stocks to 50 ml Falcon tubes (Greiner BioOne, Alphen aan den Rijn, The Netherlands) containing 5 ml of BHI and incubating the cultures for 16 -18 h (h). Optical density at 600 nm (OD_600_) was measured with the spectrophotometer (Amersham Bioscience, Twente, The Netherlands) and used as a measure for cell biomass.

**Table 1:** Strains and plasmids used in this study

Strain	Description	References
ATCC14579	*B. cereus* wild type strain	Laboratory collection
FM1004	*B. cereus* Δ*sigB* marker free KO mutant	This study
FM1009	*B. cereus* Δ*bc1009* marker free KO mutant	This study
FM1642	*B. cereus* Δ*flgG* marker free KO mutant	Laboratory collection
**Plasmid**	**Description**	**References**
pAUL-A	Heat-inducable suicide vector for *B. cereus* group, Erm^R^	Chakraborty et al., 1992

### Gene expression study

The differential SigB gene cluster expressions in wt, Δ*sigB,* and Δ*bc1009* cells before and after heat shock (30 °C versus an upshift from 30 °C to 42 °C) were compared via Reverse Transcription Quantification Polymerase Chain Reaction PCR (RT-qPCR) as described below.

### Cell culture and sample collection

ON cultures were used to inoculate 60 ml of fresh BHI to an OD_600_ of 0.01 and grown to the mid-exponential phase (OD_600_ ~ 0.4; time point zero, T0). A volume of 8 ml of the cell culture was collected as the control (T0), and the tube containing the remaining cultures was transferred to a 42 °C water bath. Then, 8 ml of heat-treated cultures were collected after 20 min (min). The collected cells were centrifuged at 8000 rpm for 5 min, resuspended in 1 ml of Tri-reagent (Merck, Zwijndrecht, The Netherlands), transferred to a lysing matrix B tube (MP Biomedicals, Eschwege, Germany), and stored at -80ºC. Four biological replicates were collected and analyzed as described below.

### RNA isolation

Cell samples in lysing matrix B tubes were homogenized using a Fastprep-24 beat beater (MP Biomedicals) for 30 s at 6.0 m/s. The procedure was repeated 6 times with resting intervals of 1 min. After 10 min settling at room temperature (RT), 200 μl of chloroform was added to the tubes, mixed, incubated for 15 min at RT, then centrifuged at 13,000 rpm for 15 min. The liquid phase (top layer) was transferred to a new Eppendorf tube, adjusted to 1 ml with RNAse-free water, mixed with 1 ml of ice-cold isopropanol, incubated at RT for 10 min, and centrifuged at 13,000 rpm for 15 min. The cell pellet was washed with 75% ice-cold ethanol, air-dried, and resuspended in 90 μl of RNAse-free water. The extracted RNA was treated with DNaseI to remove DNA, as described in the manufacturer’s protocol (Thermo Fischer Scientific, Bleiswijk, The Netherlands) and stored with 0.1 volume of 3 M sodium acetate (pH 5.2) and 2.5 volumes of 100% ethanol (v/v).

#### Reverse Transcription Quantification Polymerase Chain Reaction (RT-qPCR)

**Fig. 1:**
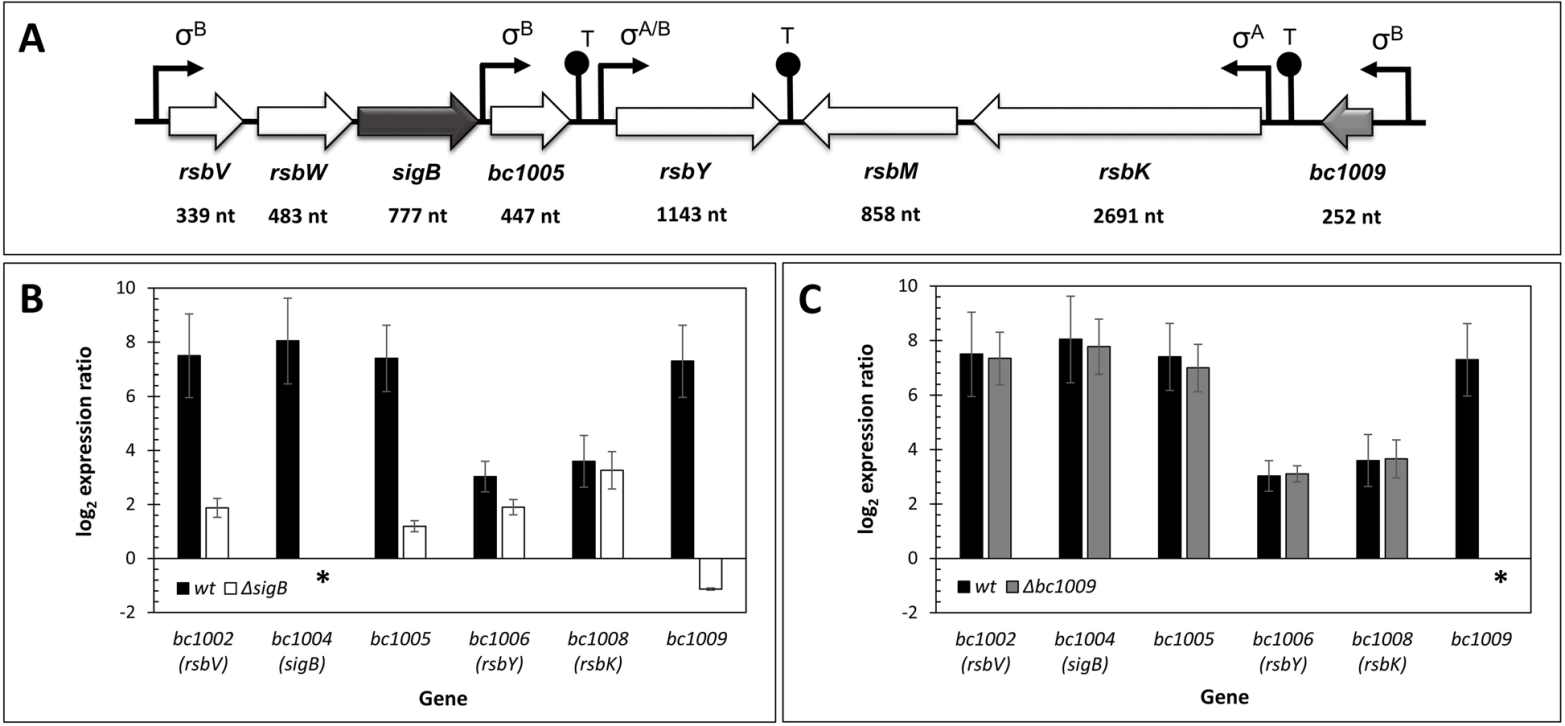
SigB gene cluster in *Bacillus cereus* and differential gene expression after heat shock. **A-** SigB gene cluster arrangement in *B. cereu*s ATCC14579. **B-** Change in expression of genes belonging to the *sigB* gene cluster for wt and Δ*sigB* mutant after 20 min of heat shock at 42 °C compared to the non-heat-stressed condition at 30 °C. *N* = 4, *p* < 0.001. **C-** Change in expression of genes belonging to the SigB gene cluster expression for wt and Δ*bc1009* mutant after heat shock at 42 °C compared to the non-heat-stressed condition at 30 °C; Black bar - wt; white bar - Δ*sigB* mutant; grey bar - Δ*bc1009* mutant. * indicates the absence of gene activity. *N* = 4, *p* < 0.001, refers to the difference in expression values of wt vs. either Δ*sigB* or Δ*bc1009* mutant

All RT-primers were designed using the Primer3 and DNASTAR software to amplify genes in the SigB gene cluster*: bc1002* (*rsbV*), *bc1003* (*rsbW*), *bc1004* (*sigB*), *bc1005*, *bc1006* (*rsbY*), *bc1008* (*rsbK*), and *bc1009* (Fig. 1A), and four reference genes *nifU, gatB, rpsU,* and *tufA*. All primers are listed in Supplementary Table S1. As *bc1007* (*rsbM*) is co-regulated with *bc1008* (*rsbK*) and does not have a SigB promoter binding motif (Fig. 1A), it was excluded from the gene expression studies.

The purified RNA samples were precipitated at 13,000 rpm for 15 min, washed with 75% ethanol, air-dried, and resuspended in 200 μl RNAse-free water. For each reaction, 600 ng of total RNA was mixed with 2 μl reverse primer (5 μM), 2 μl dNTP mix (10 mM), and added to 29 μl with RNAse-free water. The reaction mixture was incubated at 65ºC for 5 min for primer annealing and then cooled on ice for 1 min. 11 μl of reverse transcriptase master mix containing 8 μl 5 × first strand buffer, 2 μl 0.1 M DTT, and 1 μl SuperScript™ III Reverse Transcriptase (Thermo Fischer Scientific) was added to each reaction. cDNA was synthesized at 55ºC for 1 h, followed by enzyme inactivation at 70ºC for 15 min. The synthesized cDNAs were diluted tenfold, and qPCRs were performed using primers listed in Supplementary Table S1.

For qPCR, each reaction contained: 12.5 μl Power SYBR Green PCR Master Mix (Thermo Fisher Scientific), 1 μl forward primer (5 μM), 1 μl reverse primer (5 μM), 5 μl of cDNA template, and 5.5 μl of RNAse free water. DNA denaturation was done at 95ºC for 10 min, and PCRs were performed for 40 cycles (Denaturation: 95ºC, 15 secs; Annealing and elongation: 61ºC, 1 min). The cycles of quantification (Cq) values were recorded to compare differential gene expressions. Efficiencies of primers were checked with serial dilutions of the tested sample and calculated via the REST2 qPCR data analysis tool as described [33, 34]. Cq values of all tested genes were uploaded to the COTTON EST DATABASE to look for stable reference genes. *tufA, gatB,* and *rpsU* were selected [35, 36] and used to normalize the gene expression data with the REST2 qPCR program.

All recorded values were first normalized with the expression ratio of the three reference genes *gatB, rpsU,* and *tufA,* then normalized with the expression values recorded for wt in the control condition at 30 °C. The changes in gene expression of Δ*sigB* mutant vs. wt and Δ*bc1009* mutant vs. wt were expressed in log_2_ ratios.

### Transcriptome analysis

#### Cell culture and sample collection

Wt, Δ*sigB,* and Δ*bc1009* cells were cultured as described in the Cell culture and sample collection section, and RNAs were isolated and purified as mentioned in the RNA isolation section.

#### cDNA synthesis and labeling

The cDNA synthesis and labeling were performed as reported in van Schaik et al. [10] and Mols et al. [37] with slight modification. Briefly, the purified RNA was reversely transcribed using the SuperScript™ IV Reverse Transcriptase kit (Invitrogen, Groningen, the Netherlands) according to the manufacturer's instruction. mRNAs within the synthesized cDNA were treated with 3 μl of 2.5 M NaOH, spun down, incubated at 37ºC for 15 min, and neutralized with 15 μl 2 M HEPES free acid. Then, the amino-allyl-labeled dUTP cDNA was synthesized, purified with the CyScribe GFX purification kit, and labeled with the Amersham CyDye Reactive Dye (Cy3 and Cy5) (Sigma).

#### Microarray design, hybridization, and scanning

Microarray design, hybridization, and scanning were performed as described in Mols et al. [38] with slight modification. Briefly, ~ 100 ng of Cy3 and Cy5-labelled cDNA (in 1:1 ratio) were combined in a total volume of 18 µl, heated to 98ºC for 3 min, mixed with 36 μl of 2 × Hi-RPM Hybridization Buffer (Agilent Technologies, CA, USA) and hybridized onto the DNA MicroArray slides. The array slides were scanned with an Agilent G2505C scanner (Agilent Technologies).

#### Microarray data acquisition and analysis

Images were analyzed and processed with the Agilent Feature Extraction software (version 10.7.3.1). The obtained extracted data files were analyzed using the limma software package [38] in R [39]. For Agilent-based arrays, global loess normalization was used when the bulk of genes is not differentially expressed. The analysis was based on a 2-color experimental design using a linear modeling approach by lmfit and empirical Bayes statistics [40].

DNA arrays generated gene expression data for 5283 annotated coding sequences (CDSs) and 69 RNA features (sRNAs). Genes were considered as significantly differentially expressed when the transcript level ratio between the two conditions (i.e., 30 °C vs. 30 °C > 42 °C) was ≥ 2 folds (= upregulation of 1 log_2_ expression unit) or ≤ 2 folds (downregulation of 1 log_2_ expression unit) and the false discovery rate was ≤ 0.05.

### Proteome analysis

#### Cell culture and sample collection

The proteomes of wt, Δ*sigB,* and Δ*bc1009* cells grown before (30 °C) and after heat shock (42 °C) were analyzed. ON cultures of each strain were used to inoculate 80 ml of fresh BHI to an OD_600_ of 0.01, and cultures were grown to an OD_600_ ~ 0.4 at 30 °C and 200 rpm, respectively. Then, 20 ml aliquots of each culture were transferred to 50 ml Falcon tubes, and cells were spun down at 8000 rpm for 5 min at RT. After centrifugation, the cell pellet of one of the four tubes was frozen immediately in liquid nitrogen and used as the T0 control. For the remaining three tubes, cells were resuspended in 20 ml of 42 °C-preheated BHI medium and further incubated for another 40 min. Samples were harvested after 20 min (T20) and 40 min (T40), respectively, and stored at -20 °C. Four independent biological replicate experiments were performed. T20 was selected for proteome sample collection because SigB expression was shown to be the most abundant at 20 min after heat shock (data not shown), whereas T40 was selected to ensure proteins that required extended expression time were also analyzed and covered.

#### Protein extraction and preparation for mass-spectrometry analysis

The pellets were each resuspended in 100 µl Tris–HCl (5 mM pH 7.4 containing 5% SDS) and immediately disrupted using a Dismembrator (Retsch, Haan, Germany) at 2600 rpm for 3 min (in a 4,8 ml Teflon vessel precooled in liquid nitrogen with an 8 mm diameter steel ball). Next, the cell powder was resuspended with 400 µl of preheated (95 °C) Tris–HCl buffer (5 mM pH 7.4) and the viscous lysate was transferred into a fresh 1.5 ml low bind pre-lubricated Eppendorf tube and shaken for 1 min at 95 °C and 1400 rpm. The lysate was cooled to RT and 2 µl of a 1 M MgCl_2_ stock solution (final 4 mM MgCl_2_) was added. Next, 1 µl of a 1:100 diluted benzonase (Pierce Universal Nuclease No#88,702) (Pierce, Rockford, IL, USA) stock solution (final 0.005 U/µl) was added and mixed by short vortexing. The samples were incubated in an ultrasonic bath at RT for 5 min until the viscous lysate was liquefied by complete degradation of DNA and RNA. The raw lysates were centrifuged at 17,000 g at RT for 30 min. After centrifugation, the protein lysate was transferred into a fresh 1.5 ml low bind pre-lubricated Eppendorf tube, and the pelleted cell debris was discarded. The protein concentration of the samples was determined using the Micro BCA Protein Assay Kit following the manufacturer’s protocol (Pierce, Prod. No. 23235) using a FLUOstar Omega Plate Reader (BMG Labtech, Ortenberg, Germany). Samples were stored at -80 °C. Sample preparation for mass spectrometry measurements was performed using the SP3 protocol as described in Blankenburg et al. [41].

#### Liquid chromatography-mass spectrometry (LC–MS) measurements

Protein samples were measured using the LC–MS/MS platform containing reversed-phase nano liquid chromatography (nano Acquity M-class UPLC) (Waters Corporation, Massachusetts, USA), which was coupled to nanospray ionization tandem mass spectrometry with traveling wave ion mobility using high-definition data-independent (HD-MS^E^) acquisition and enabled with hybrid quadrupole orthogonal acceleration time of flight mass spectrometer (Synapt G2Si, Waters Corporation).

The peptide mixture was separated on ACQUITY UPLC® M-Class HSS T3 1.8 um, 75 um × 200 mm column (Waters Corporation) using a mixture of Buffers A (0.1% v/v Acetic acid in water) and B (0.1% v/v acetic acid in acetonitrile) through a linear concentration gradient of Buffer B in 170 min, with a flow rate of 300 nl/min from 5–26% (v/v). The eluents were sprayed at a voltage of 1.85–1.90 kV using PicoTip emitters (Waters Corporation), and other source parameters were set (sampling cone = 40 V, source offset = 0 V, source temperature = 80 °C, gas flows, cone gas = 50 l/h, nano gas flow = 0.4 bar, purge gas = unchanged, IMS = optimized for wave velocity with a start velocity of 870 m/s to end at 564 m/s that corresponds to the separation of GluFib fragments in the drift time range of 0–200 bins).

The data was acquired using the MassLynx™ software program version V4.1 (Waters Corporation) that automatically switches between MS and MS/MS (HDMS^E^) scans in resolution mode at 20,000 with 1 s (sec) scan time and the scan range of 50–2000 m/z. Calibration was done by injecting GluFib at an interval of 1 min. The acquired data were analyzed for protein identifications against the Uniprot fasta database of *B. cereus* ATCC14579 strain that contained 5240 sequences via the PLGS v3.3 program (Water Corporation). Spectra were processed using low and high-energy thresholds of 135, 20 counts, and lock mass calibration 785.8456 m/z for GluFib. The workflow search parameters were set as: (protease = trypsin, one missed cleavage, carbamidomethyl for cysteine as fixed, variable modifier = oxidation of methionine). The protein was quantified when 1) the top 3 peptides had no modifications; 2) pass one match had peptide fragment one; and 3) ranked the first three highest peptides. The PLGS independent identification output was imported to ISOQuant 1.8 [42] for sample analyses.

#### Proteome data analysis

The quantitative protein data were obtained based on identifying a minimum of two peptides, the protein areas were used, and the data analysis was performed using the tidyverse package (version 1.3.0) in R version 4.0.2 [43]. Briefly, the IsoQuant protein intensities were median normalized using the global median as reference. The PCA analysis was carried out using the FactoMineR package (version 2.3) [44] with normalized log_2_ protein intensities scaled to unit variance. The sample correlation was calculated using Kendall's methodology [45] and displayed using the ggcorrplot package (version 0.1.3) [46]. The statistical analysis was carried out using the PECA package (version 1.24.0) [47] by applying a modified T-test for the pairwise comparisons to proteins having valid protein quantity values in at least two replicates, by calculating empirical Bayes moderated T-statistics using the linear modeling approach implemented in the limma package (version 3.44.3) [38]. The raw *p*-values (p) were multiple tests adjusted (p.fdr) using the Benjamini–Hochberg method [48]. Finally, volcano plots were generated using the ggplot2 package (version 3.3.2) [49] using an absolute fold-change cut-off of 1.5 and 0.05 as q-value (adjusted *p*-value) cut-off.

Mass spectrometric analyses detected between ~ 1100 to ~ 1800 proteins, depending on the strain and condition. Proteins were considered to be significantly differentially expressed when the absolute fold change (FC) was ≥ 1.5 folds (log_2_ FC ≥ 0.585) (induction/upregulation) or ≤ 1.5 folds (log_2_ FC ≤ 0.585) (downregulation), and the false discovery rate was ≤ 0.05.

### Cell survival study

ON cultures were inoculated into 60 ml of fresh BHI to an OD_600_ of 0.01 and grown to the mid-exponential phase (OD_600_ ~ 0.4, T0) at 30 °C. A control sample (T0 _non-adapted_) was collected, and the remaining cultures were divided into two portions. One portion was heated at 50 °C without preadaptation, and samples were collected after 10, 20, and 30 min. Another portion was preadapted at 42 °C for 45 min, a control sample (T0 _adapted_) was collected, and the tube with the remaining culture was heated at 50 °C. Samples were taken every 20 min intervals up to 120 min. Preadaptation for 45 min at 42 °C was chosen to allow an adequate time for the culture medium to reach 42 °C, as a 15—60 min preheating treatment showed similar effects upon exposure to this lethal temperature of 50 °C [5].

At every time point, cultures were tenfold serially diluted and plated on BHI agar plates. Colony-forming units per ml (CFU)/ml were calculated, and the survival of the heat-adapted and non-heat-adapted cells was expressed in log_10_ CFU/ml reduction (log_10_ CFU/ml T_0_—log_10_ CFU/ml T_x_). The experiment was done with biological and technical duplicates, respectively.

### Cell motility assay of wt, Δ*sigB*, and Δ*bc1009* cells

A cell motility assay was performed to check the phenotype for the wt, Δ*sigB,* and Δ*bc1009* cells at the control temperature (30 °C) and under the condition with heat stress (30 °C → 42 °C). A flagella deficient strain Δ*flgG* (laboratory collection) was used as the negative control. The motility of each strain was tested on a BHI medium with 0.25% agar. Plates were prepared by pouring 50 ml in 120 × 18 mm square Petri dishes (Greiner Bio-one). The BHI plates were dried for 15 min in a flow cabinet before use.

All strains were cultured ON at 30 °C, 200 rpm. An aliquot of 250 μl of an ON culture was used to inoculate 20 ml of fresh BHI medium, and the culture was grown to an OD_600_ of ~ 0.4. Of these cultures, cells were collected from 5 ml via centrifugation at 5500 g for 5 min. The supernatant was discarded, and the cell pellet was resuspended in 5 ml Phosphate-buffered saline (PBS). The motility under heat stress was assessed by first subjecting 5 ml of the cultures to 42 °C in a water bath for 30 min, followed by centrifugation and resuspension in PBS to standardize initial inoculation levels and prevent carry over of compounds in the culture medium that might affect the motility assay on various types of media. 5 µl of the resuspended cells were pipetted in the middle of the BHI agar plates, dried in the flow cabinet, and incubated either at 30 °C or 42 °C. The closed agar Petri dishes were covered with wet tissues to prevent dehydration. After ~ 20 h, all plates were photographed, and the colony diameter was measured using the ImageJ software [50]. The colony diameter and maximum plate size in pixels were measured in ImageJ with the ‘Rectangle’ setting. The colony diameter (in pixels) was divided by the maximum plate size (in pixels) to obtain a colony/plate ratio. This ratio was subsequently multiplied by the known plate size (150 mm) to obtain the colony diameter in mm.

## Results

The *bc1009* gene in the SigB gene cluster (Fig. 1A) encodes a putative Hpr-like phosphocarrier protein (Bc1009) in *B. cereus.* The potential role of *bc1009* acting as a SigB regulator was first examined via gene expression studies, and its SigB-dependent functions were investigated via proteome profiling (complemented with transcriptomic data). The global protein/gene expressions of the Δ*bc1009* mutant before and after heat shock were analyzed and compared with the protein/gene expressions of the *B. cereus* wt cells and a marker-free Δ*sigB* mutant under the same conditions.

### Expression of *bc1009* is dependent on *sigB*, but deletion of *bc1009* does not affect the expression of *sigB*

Changes in expression within the SigB gene cluster*: bc1002* (*rsbV*), *bc1003* (*rsbW*), *bc1004* (*sigB*), *bc1005*, *bc1006* (*rsbY*), *bc1008* (*rsbK*), and *bc1009* of *B. cereus* wt, Δ*sigB,* and Δ*bc1009* cells upon heat shock (30 °C → 42 °C) were determined via RT-qPCR. Results were first compared for wt versus the Δ*sigB* mutant (Fig. 1B), and then for wt versus the Δ*bc1009* mutant (Fig. 1C).

Upon heat shock of wt cells, the expression of the three known SigB-dependent genes *rsbV* (*bc1002*), *bc1005*, *bc1009,* and *sigB* itself increased > 200-fold (~ log_2_ 8) compared to wt cells without heat shock. The *rsbY* (*bc1006*) and *rsbK* (*bc1008*) genes that encode the two-component system were also expressed ~ 4- fold (~ log_2_ 2) higher in wt cells after heat shock than before heat shock (Figs. 1B and C). Upon heat shock of the Δ*sigB* mutant, *rsbV* and *bc1005* were mildly expressed (< 4- fold) (~ log_2_ 2), whereas *bc1009* was expressed at a lower level after heat shock in the Δ*sigB* mutant (Fig. 1B), showing that its heat induction was dependent on SigB. The expression of the histidine kinase encoding gene *rsbK* is controlled by *sigA.* The observation that its expression was similar for the Δ*sigB* mutant and the wt cultures after heat shock is in line with this. Its cognate regulator partner *rsbY* was expressed at a lower level in the Δ*sigB* mutant than in wt cultures, indicating partial dependency of its expression on SigB (Fig. 1B)*.* Notably, when the *bc1009* gene was deleted, the expression of all other genes in the SigB cluster was unaffected and similar to that in wt cultures (Fig. 1C). This implied that *sigB* expression did not rely on *bc1009*, ruling out a possible role of *bc1009* as an additional phosphocarrier in the activation of SigB via the RsbKY system.

### Comparison of proteomic profiles of Δ*sigB *and Δ*bc1009* mutants to wt upon heat shock reveals novel SigB-dependent proteins conceivably mediated via Bc1009 in *B. cereus*

To further explore the role of Bc1009 and SigB in *B. cereus,* we compared the proteome profiles of *B. cereus* cultures of wt, Δ*sigB,* and Δ*bc1009* mutants grown at 30 °C with cultures that were heat-shocked at 42ºC after growth at 30 °C. The proteomics results of wt, Δ*sigB,* and Δ*bc1009* cells obtained in this study are presented below and complemented by transcriptomics results (Supplementary files).

#### Heat-induced changes of proteome profiles in wt cells

**Fig. 2:**
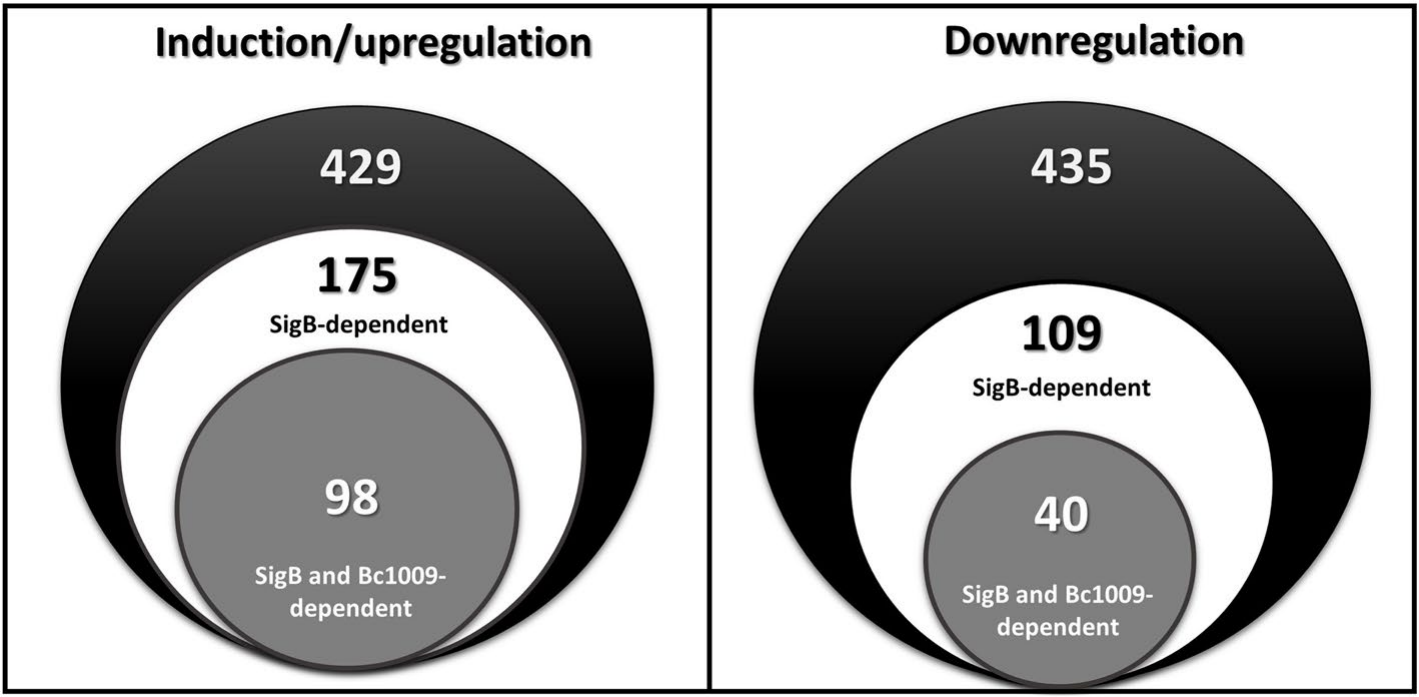
Proteomics analyses of *B. cereus* ATCC14579 wt, Δ*sigB,* and Δ*bc1009* mutants upon heat shock. Left: Venn diagram showing 429 significantly induced (upregulated) proteins in *B. cereus* heat-stressed wt cells (black circle) compared to the non-heat-stressed wt cells at 30 °C (see Supplementary Table S2A). White circle: 175 heat-induced proteins in wt that are SigB-dependent, i.e., proteins that show upregulation of > 0.6 log_2_ fold change in wt/*ΔsigB* cells upon heat shock compared to the non-heat-stressed condition at 30 °C. Grey circle: For 98 proteins, the heat-mediated increase in level in the wt is dependent on SigB and Bc1009, i.e., proteins that show upregulation of > 0.6 log_2_ fold change in wt/Δ*sigB* and wt/Δ*bc1009* cells upon heat shock compared to the non-heat-stressed condition at 30 °C (see Table 2 for the complete list of proteins). Right: Venn diagram showing 435 significantly downregulated proteins in *B. cereus* heat-stressed wt cells (black circle) compared to the non-heat-stressed wt cells at 30 °C (Supplementary Table S2A). White circle: 109 downregulated proteins in heat-stressed wt cells vs. non-heat-stressed wt cells are SigB-dependent, i.e., proteins that show downregulation of > 0.6 log_2_ fold change in wt/Δ*sigB* cells upon heat shock compared to the non-heat-stressed condition at 30 °C. Grey circle: For 40 proteins, reduction in level in wt cells upon heat shock was dependent on SigB and Bc1009, i.e., proteins that show downregulation of > 0.6 log_2_ fold change in wt/Δ*sigB* and wt/Δ*bc1009* cells upon heat shock vs. non-heat-stressed condition (see Table 3). The underlying transcriptome data supporting this figure are presented in Supplementary Table S2B

After heat shock treatment, ~ 1500 proteins were detected for wt cells. Among these, 429 proteins displayed significantly increased (≥ 1.5 folds, log_2_ FC ≥ 0.585), and 435 significantly decreased (≤ 1.5 folds, log_2_ FC ≤ 0.585) levels in wt cells after heat shock compared to control conditions, respectively (Fig. 2). The complete list of proteins displaying significantly different levels is presented in Supplementary Table S2A, and the clusters of orthologous group (COG) functions are presented in Supplementary Figure S1.

As expected, next to previously described SigB regulon members (see below), well-known protein chaperones (GroEL, DnaJ, DnaK), transcription repressors (HrcA, CtsR) that control Clp proteases (ClpC, ClpP, ClpB, ClpY, ClpQ), DNA repair proteins (RadA, MutS) and other well-known heat shock proteins like GrpE, YflT, and FtsH were all present at ≥ 1.5 folds higher levels in wt cultures after heat shock at 42 °C compared to control conditions at 30 °C. These heat shock proteins were also identified in an earlier study on *B. cereus* heat stress response [51]*,* confirming the congruency of the results obtained in this study. Extended information on the *B. cereus* heat shock regulon was acquired (Supplementary Table S2A), and ~ 20% of the proteins with temperature-dependent changes in level found in wt cultures upon heat shock were also influenced at the gene expression level (Supplementary Table S2B).

#### Impact of SigB and Bc1009 on *B. cereus* protein profiles after heat shock

##### SigB-dependent and SigB/Bc1009-dependent heat shock induction of proteins

**Fig. 3:**
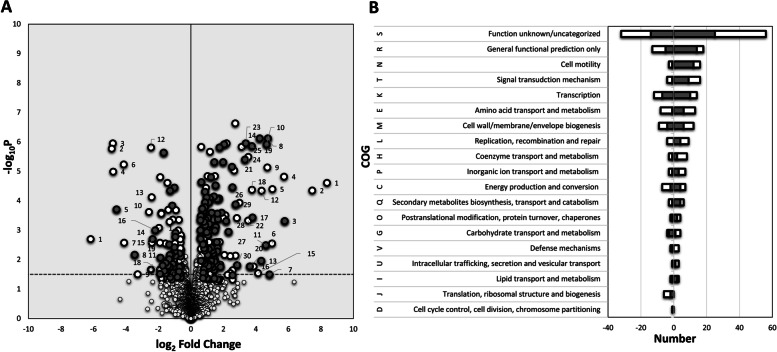
SigB and SigB (and Bc1009)-dependent induced and downregulated proteins in wt cells after heat shock (30 °C to 42 °C) versus before heat shock and their cluster of orthologous group (COG). **A**- log_2_ fold change in protein expression. Positive and negative fold change values indicate induced and downregulated proteins, respectively. Numbers indicate the top 30 induced (Table 2) or top 20 downregulated (Table 3) proteins, respectively. The *p*-value threshold for each protein was < 0.05, *N* = 4. Grey symbols: induced/downregulated proteins that are SigB (and Bc1009-dependent; i.e., proteins that show up(or down) regulation of > 0.6 log_2_ fold change in wt/Δ*sigB* and wt/Δ*bc1009* cells upon heat shock vs. non-heat-stressed condition. White symbols: induced/downregulated proteins that are SigB-dependent; i.e., proteins that show up(or down) regulation of > 0.6 log_2_ fold change in wt/Δ*sigB* cells upon heat shock vs. non-heat-stressed condition. **B**- cluster of orthologous group (COG) function for both SigB-dependent induced (Right) and downregulated proteins (Left) in wt cells upon heat shock. The number on the x-axis indicates the total number of induced/downregulated proteins, and the negative sign indicates downregulation. Grey bar: induced/downregulated proteins after heat shock that are SigB (and Bc1009)-dependent, i.e., proteins that show up(or down) regulation of > 0.6 log_2_ fold change in wt/*ΔsigB* and wt/Δ*bc1009* cells; White bar: induced/downregulated proteins that are SigB-dependent, i.e., proteins that show up(or down) regulation of > 0.6 log_2_ fold change only in wt/Δ*sigB* cells respectively, compared to the expression in the non-heat-stressed condition at 30 °C. The underlying transcriptome data supporting this figure are presented in Supplementary Table S3B

The protein profiles of Δ*sigB* and Δ*bc1009* mutants were further compared with wt cells. Out of 429 proteins displaying increased levels after heat shock in wt cells, 175 showed lower levels in the Δ*sigB* mutant, indicating that the expression of the encoding genes was inducible via SigB in wt cells directly or indirectly (Fig. 2, Table 2). Additionally, for 98 of these 175 proteins, this effect was not only dependent on SigB but also on Bc1009 because they also exhibited lower levels in the Δ*bc1009* mutant than in the wt (Fig. 2, Table 2), hereby referred to as SigB (and Bc1009)-dependent proteins. The complete list of the SigB-induced and SigB (and Bc1009)-induced proteins with their functional annotation is listed in Table 2. The log_2_ fold changes of the protein levels are presented in the volcano plot in Fig. 3A, and their COG functions in Fig. 3B. The top 30 most significant SigB-induced proteins are indicated with numbers in Fig. 3A on the right (marked in white), and for half of these, the increase in level was also dependent on Bc1009 (marked in Grey in Fig. 3A) (see details in Table 2).

**Table 2:** SigB-dependent induced proteins upon heat shock in *B. cereus* ATCC14579^**a**^

No	Locus Tag	Protein	Annotation	wt 42 °C/ Δ*sigB* 42 °C log_2_ FC	wt 42 °C/ Δ*bc1009* 42 °C log_2_ FC	COG
1	BC0107	YacN	2-C-methyl-D-erythritol 2,4-cyclodiphosphate synthase	8.4		I
2	BC0998^b^	YflT	General stress protein 17 M	7.5		S
**3**	**BC4345**	**BC4345**	**Lipase; Pimeloyl-ACP methyl ester carboxylesterase (Coenzyme transport & metabolism)**	**5.8**	**5.5**	**R**
4	BC1004 ^b^	SigB	RNA polymerase sigma-B factor	5.7		K
5	BC1005 ^b^	BC1005	Bacterioferritin	5.0		P
6	BC4336	SigK	RNA polymerase sigma-K factor, sporulation sigma factor	5.0		K
**7**	**BC0409**	**BC0409**	**Carbamate kinase**	**4.8**	**1.5**	**E**
**8**	**BC5066**	**BC5066**	**Endonuclease/Exonuclease/phosphatase family protein**	**4.7**	**4.2**	**S**
9	BC0863 ^b^	KatE	Catalase	4.7		P
**10**	**BC0666**	**BC0666**	**Immune inhibitor A precursor**	**4.7**	**3.7**	**S**
**11**	**BC0862** ^b^	**YfkM**	**Protease I**	**4.6**	**1.1**	**R**
12	BC4153	BC4153	Phosphohydrolase (MutT/nudix family protein)	4.3		L
**13**	**BC3935**	**BC3935**	**hypothetical Cytosolic Protein**	**4.3**	**4.1**	**S**
**14**	**BC1010** ^b^	**BC1010**	**YflT heat induced stress protein family**	**4.2**	**0.7**	**S**
15	BC0422	BC0422	Methyl-accepting chemotaxis protein	4.1		NT
16	BC0612	BC0612	L-lactate permease	3.9		C
**17**	**BC1663**	**FliN**	**Flagellar motor switch protein fliN**	**3.8**	**3.6**	**NU**
18	BC0613	BC0613	Transcriptional regulator, ArsR family	3.8		K
**19**	**BC5077**	**BC5077**	**Protein with unknown function**	**3.7**	**3.8**	**S**
**20**	**BC3110**	**YndF**	**Spore germination protein BC**	**3.6**	**1.2**	**S**
21	BC3229	YxkD	hypothetical Membrane Spanning Protein	3.5		S
22	BC0773	YdjE	Fructokinase	3.5		G
**23**	**BC3576**	**BC3576**	**Spore germination protein SC**	**3.4**	**3.2**	**S**
**24**	**BC5410**	**YocJ**	**FMN-dependent NADH-azoreductase**	**3.3**	**5.2**	**I**
25	BC0999 ^b^	BC0999	General stress protein (hyperosmotic & cold)	3.1		S
26	BC3292	BC3292	hypothetical protein	3.0		S
**27**	**BC1662**	**FliM**	**Flagellar motor switch protein fliM**	**2.8**	**2.9**	**N**
28	BC1661	BC1661	Flagellar motor switch protein fliN	2.8		NU
**29**	**BC3406**	**BC3406**	**Oxidoreductase**	**2.8**	**3.5**	**R**
30	BC1127	BC1127	Malate synthase	2.7		C
31	BC1002 ^b^	RsbV	Anti-sigma B factor antagonist	2.7		T
32	BC1003 ^b^	RsbW	Anti-sigma B factor	2.7		T
33	BC1025	BC1025	Glyoxalase family protein	2.6		E
34	BC3132 ^b^	BC3132	General stress protein 17 M	2.6		S
**35**	**BC0423**	**SrfAAH**	**Non-ribosomal peptide synthetase (adenylation domain)**	**2.6**	**2.6**	**Q**
**36**	**BC2006**	**TlpA**	**Methyl-accepting chemotaxis protein, pH sensor**	**2.6**	**2.6**	**NT**
**37**	**BC5422**	**BC5243**	**O-Antigen ligase—like protein**	**2.5**	**2.9**	**S**
38	BC1226	YjcH	Acetyl esterase	2.4		P
39	BC0655	BC0655	Universal stress protein family	2.4		T
**40**	**BC5436**	**BC5436**	**Peptide methionine sulfoxide reductase**	**2.3**	**2.4**	**O**
41	BC1237	TrpB	Tryptophan synthase beta chain	2.3		E
42	BC1333	BC1333	CBS domain containing protein	2.2		R
**43**	**BC1349**	**BC1349**	**Acetyltransferase**	**2.2**	**0.8**	**KR**
44	BC1369	DltD	Protein dltD precursor	2.1		M
45	BC1403	LeuD	3-isopropylmalate dehydratase small subunit	2.1		E
**46**	**BC1303**	**YvfV**	**(S)-2-hydroxy-acid oxidase, iron-sulfur chain**	**2.0**	**2.1**	**C**
47	BC4463	BC4463	Stage II sporulation protein B	2.0		S
**48**	**BC3980**	**YkuR**	**putative N-acetyldiaminopimelate deacetylase, peptidoglycan synthesis**	**2.0**	**1.9**	**R**
**49**	**BC4786**	**BC4786**	**hypothetical Cytosolic Protein**	**1.9**	**4.8**	**S**
**50**	**BC1410**	**HisF**	**HisF protein**	**1.8**	**1.7**	**E**
51	BC5223	YdaJ	hypothetical protein	1.8		S
52	BC1431	BC1431	Cell wall endopeptidase, family M23/M37	1.8		MS
53	BC4820	BC4820	hypothetical protein	1.8		S
**54**	**BC5141**	**CggR**	**Central glycolytic genes regulator**	**1.8**	**1.7**	**K**
**55**	**BC1370**	**DltC**	**D-alanyl carrier protein**	**1.7**	**2.9**	**IQ**
**56**	**BC0678**	**BC0678**	**Methyl-accepting chemotaxis protein, signaling domain**	**1.7**	**1.4**	**NT**
**57**	**BC5123**	**BC5123**	**homolog of lantibiotic biosynthesis dehydratase C-term**	**1.7**	**1.7**	**S**
**58**	**BC0597**	**YueK**	**Nicotinate phosphoribosyltransferase**	**1.7**	**1.7**	**H**
**59**	**BC5118**	**BC5118**	**ABC transporter ATP-binding protein**	**1.6**	**1.8**	**E**
**60**	**BC1275**	**BC1275**	**Methyltransferase**	**1.6**	**1.6**	**QR**
**61**	**BC5155**	**YvcK**	**hypothetical Cytosolic Protein**	**1.6**	**2.2**	**S**
**62**	**BC5103**	**YclP**	**Ferric anguibactin transport ATP-binding protein**	**1.6**	**2.0**	**P**
**63**	**BC3203**	**BC3203**	**hypothetical protein**	**1.6**	**1.5**	**S**
64	BC2303	DhbC	Isochorismate synthase	1.6		HQ
**65**	**BC3257**	**BC3257**	**N-acetylmuramoyl-L-alanine amidase, Cell wall or membrane biogenesis**	**1.5**	**1.6**	**TM**
**66**	**BC5122**	**BC5122**	**YcaO cyclodehydratase, ATP-ad Mg2 + -binding**	**1.5**	**1.8**	**S**
67	BC0571	BC0571	Serine/threonine protein phosphatase	1.5		T
**68**	**BC0675**	**BC0675**	**hypothetical protein**	**1.5**	**1.7**	**S**
69	BC1586	TuaA	Undecaprenyl-phosphate galactosephosphotransferase	1.5		M
70	BC3350	BC3350	TPR-repeat-containing protein	1.5		R
**71**	**BC2850**	**YkfB**	**Mandelate racemase/muconate lactonizing enzyme family protein**	**1.5**	**1.1**	**MR**
**72**	**BC3165**	**PucA**	**Xanthine dehydrogenase subunit**	**1.5**	**1.0**	**O**
73	BC3287	BC3287	hypothetical protein	1.4		S
**74**	**BC5255**	**YknX**	**periplasmic component of efflux system**	**1.4**	**1.6**	**M**
**75**	**BC1857**	**BC1857**	**Exonuclease SbcD**	**1.4**	**1.8**	**L**
76	BC3278	BC3278	hypothetical protein	1.4		S
**77**	**BC1658**	**YvzB**	**Flagellin**	**1.4**	**1.3**	**N**
**78**	**BC2757**	**BC2757**	**Tryptophan 2,3-dioxygenase**	**1.4**	**2.4**	**E**
79	BC5125	BC5125	Peptidase family M50	1.4		M
80	BC0419	ThiM	Hydroxyethylthiazole kinase	1.4		H
81	BC1725	DsdA	D-serine dehydratase	1.4		E
**82**	**BC1756**	**PadR**	**Transcriptional repressor PadR**	**1.4**	**1.0**	**K**
**83**	**BC5168**	**UvrB**	**Excinuclease ABC subunit B**	**1.3**	**1.4**	**L**
**84**	**BC5243**	**BC5243**	**hypothetical protein**	**1.3**	**1.6**	**S**
**85**	**BC5119**	**BC5119**	**In operon with BC5120, which is a nitroreductase family protein**	**1.3**	**1.6**	**S**
**86**	**BC0244**	**AppD**	**Oligopeptide transport ATP-binding protein oppD**	**1.3**	**0.7**	**EP**
**87**	**BC2147**	**RapG**	**Response regulator aspartate phosphatase**	**1.3**	**1.0**	**R**
**88**	**BC1637**	**BC1637**	**Flagellar hook-associated protein 3**	**1.2**	**1.0**	**N**
**89**	**BC4300**	**YqfG**	**hypothetical Metal-Binding Protein**	**1.2**	**1.3**	**R**
**90**	**BC2285**	**MmgD**	**2-methylcitrate synthase**	**1.2**	**1.5**	**C**
91	BC2431	BC2431	PhnB protein	1.2		S
**92**	**BC1651**	**FlgE**	**Flagellar hook protein flgE**	**1.2**	**1.1**	**N**
93	BC2425	BC2425	hypothetical protein	1.2		S
94	BC2464	BC2464	S-layer protein / Peptidoglycan endo-beta-N-acetylglucosaminidase	1.2		G
95	BC2750	BC2750	Protein with unknown function	1.2		L
**96**	**BC0792**	**YrkD**	**hypothetical Cytosolic Protein**	**1.2**	**1.4**	**S**
**97**	**BC4170**	**SpoOA**	**Stage 0 sporulation protein A**	**1.1**	**1.3**	**TK**
**98**	**BC3356**	**BC3356**	**Transcriptional regulator, MerR family**	**1.1**	**0.9**	**K**
99	BC3565	BC3565	hypothetical protein	1.1		H
**100**	**BC2760**	**BC2760**	**Transcriptional regulator, TetR family**	**1.1**	**1.1**	**K**
**101**	**BC3852**	**YlpC**	**PaaI family protein, possible transcriptional regulator**	**1.1**	**0.7**	**Q**
**102**	**BC4791**	**YtiB**	**Carbonic anhydrase**	**1.1**	**1.0**	**P**
103	BC2862	PrsA	Protein export protein prsA precursor	1.1		O
**104**	**BC4513**	**MotA**	**Chemotaxis motA protein**	**1.1**	**1.0**	**N**
**105**	**BC3894**	**BC3894**	**DnaK suppressor protein**	**1.1**	**1.3**	**T**
**106**	**BC4371**	**BC4371**	**hypothetical protein**	**1.1**	**0.9**	**S**
107	BC2919	YokD	Aminoglycoside N3'-acetyltransferase	1.1		V
**108**	**BC5121**	**BC5121**	**hypothetical protein**	**1.1**	**1.6**	**S**
**109**	**BC5117**	**BC5117**	**ABC transporter permease protein**	**1.1**	**1.2**	**S**
110	BC2936	YdgH	Transcriptional repressor Bm3R1	1.0		K
**111**	**BC3433**	**BC3433**	**hypothetical protein**	**1.0**	**1.8**	**S**
112	BC3070	BC3070	Signal peptidase I	1.0		U
113	BC0887	BC0887	Collagen adhesion protein	1.0		M
**114**	**BC3192**	**BC3192**	**precursor of the glucomannan utilization protein ydhR**	**1.0**	**2.8**	**S**
**115**	**BC0191**	**BC0191**	**hypothetical Membrane Spanning Protein**	**1.0**	**1.2**	**S**
116	BC1645	FliG	Flagellar motor switch protein fliG	1.0		N
**117**	**BC5241**	**YvbJ**	**IG hypothetical 16,680**	**1.0**	**1.1**	**S**
118	BC4938	YutJ	NADH dehydrogenase	1.0		C
**119**	**BC0954**	**BC0954**	**tcdA-E operon negative regulator**	**1.0**	**1.0**	**S**
120	BC4723	BC4723	Molybdopterin biosynthesis MoeB protein	0.9		H
121	BC3264	BC3264	hypothetical protein	0.9		L
**122**	**BC2150**	**YpgQ**	**metal-dependent phosphohydrolase**	**0.9**	**1.0**	**R**
**123**	**BC2846**	**BC2846**	**Protein dltD precursor**	**0.9**	**1.2**	**M**
**124**	**BC3281**	**BC3281**	**hypothetical protein**	**0.9**	**2.0**	**S**
**125**	**BC0404**	**TarH**	**Methyl-accepting chemotaxis protein**	**0.9**	**0.7**	**NT**
**126**	**BC1657**	**BC1657**	**Flagellin**	**0.9**	**1.1**	**S**
127	BC1882	BC1882	Phage protein	0.9		S
**128**	**BC1247**	**BC1247**	**hypothetical protein**	**0.9**	**1.1**	**S**
129	BC0295	GroEL	60 kDa chaperonin GROEL	0.9		O
**130**	**BC5120**	**BC5120**	**hypothetical Cytosolic Protein**	**0.9**	**1.7**	**S**
**131**	**BC3286**	**BC3286**	**hypothetical protein**	**0.9**	**0.8**	**C**
132	BC1384	YubB	Bacitracin resistance protein (Putative undecaprenol kinase)	0.9		V
133	BC0040	YabB	Methyltransferase	0.9		R
134	BC3550	BC3550	Argininosuccinate lyase	0.9		E
**135**	**BC1302**	**YvfI**	**Transcriptional regulator, GntR family**	**0.9**	**0.8**	**K**
**136**	**BC3601**	**YclJ**	**Two-component response regulator**	**0.9**	**0.9**	**TK**
**137**	**BC3699**	**BC3699**	**Antigen/ lysozyme like protein**	**0.8**	**3.1**	**M**
**138**	**BC0061**	**YabN**	**MazG protein**	**0.8**	**0.9**	**R**
139	BC3802	YmxH	hypothetical protein	0.8		S
**140**	**BC1924**	**BC1924**	**L-lactate dehydrogenase**	**0.8**	**1.0**	**C**
141	BC4178	BC4178	Exodeoxyribonuclease VII small subunit	0.8		E
142	BC1736	YfiN	Export ABC transporter permease protein	0.8		V
**143**	**BC4224**	**BC4224**	**Glycine dehydrogenase [decarboxylating]**	**0.8**	**1.8**	**E**
144	BC5445	BC5445	Superoxide dismutase [Mn]	0.8		P
**145**	**BC4696**	**YtmQ**	**SAM-dependent methyltransferase**	**0.8**	**1.7**	**R**
146	BC4246	BC4246	hypothetical protein	0.8		S
**147**	**BC4469**	**HemB**	**Delta-aminolevulinic acid dehydratase**	**0.8**	**0.7**	**H**
148	BC1628	CheA	Chemotaxis protein cheA	0.8		NT
149	BC4702	YtjP	Xaa-His dipeptidase	0.8		E
150	BC4261	BC4261	hypothetical Cytosolic Protein	0.8		S
151	BC1479	YvqK	hypothetical Cytosolic Protein	0.8		S
**152**	**BC0888**	**CwlH**	**N-acetylmuramoyl-L-alanine amidase,Cell wall or membrane biogenesis**	**0.8**	**0.7**	**M**
**153**	**BC0576**	**McpBH**	**Methyl-accepting chemotaxis protein**	**0.8**	**1.9**	**NT**
154	BC4381	YrrK	hypothetical Cytosolic Protein	0.7		L
**155**	**BC3921**	**YlbP**	**Acetyltransferase**	**0.7**	**0.9**	**KR**
**156**	**BC1654**	**CheV**	**Chemotaxis protein cheV**	**0.7**	**0.8**	**NT**
**157**	**BC4579**	**DnaI**	**Primosomal protein dnaI, DNA replication**	**0.7**	**2.2**	**L**
**158**	**BC4644**	**BC4644**	**PhnB protein**	**0.7**	**1.0**	**S**
159	BC3976	BC3976	putative transcriptional regulator	0.7		S
160	BC4668	BC4668	Virulence factor mviM	0.7		R
**161**	**BC4741**	**BC4741**	**DNA integration/recombination/invertion protein**	**0.7**	**0.6**	**L**
162	BC3857	YloS	Thiamin pyrophosphokinase	0.7		H
**163**	**BC4832**	**YdeE**	**Transcriptional regulator, AraC family**	**0.6**	**1.3**	**KS**
164	BC4847	BC4847	D-alanyl-D-alanine carboxypeptidase	0.6		MS
**165**	**BC1659**	**BC1659**	**hypothetical protein**	**0.6**	**0.6**	**S**
**166**	**BC3713**	**YmaH**	**Hfq protein**	**0.6**	**0.6**	**R**
167	BC4961	YutE	hypothetical Cytosolic Protein	0.6		S
168	BC4856	MenF	Isochorismate synthase	0.6		HQ
**169**	**BC3372**	**YqeC**	**6-phosphogluconate dehydrogenase**	**0.6**	**0.6**	**G**
170	BC0570	BC0570	Glycerol-3-phosphate-binding protein	0.6		G
**171**	**BC0203**	**BC0203**	**hypothetical protein**	**0.6**	**0.7**	**S**
172	BC1313	BC1313	PhaP protein	0.6		S
173	BC4179	YqiB	Exodeoxyribonuclease VII large subunit	0.6		L
**174**	**BC5034**	**YoaH**	**Methyl-accepting chemotaxis protein**	**0.6**	**0.7**	**NT**
**175**	**BC5124**	**BC5124**	**Protein with unknown function**	**0.6**	**1.9**	**S**

^a^Bold: SigB and Bc1009-dependent-induced proteins upon heat shock in *B. cereus*

Not Bold: SigB-dependent-induced proteins upon heat shock in *B. cereus*

log_2_ FC: log_2_ protein fold change values at either T20 or T40 timepoint

COG: Cluster of orthologous groups, see description in Fig. 3

^b^Indicates the presence of SigB promoter binding motif

Only SigB and Bc1009-dependent proteins are listed here

The 175 proteins displaying SigB-dependent increases in level after heat shock include the general stress defense proteins previously described by van Schaik et al. [10] and De Been et al. [12], i.e., Bc0861, Bc0862 (YfkM), Bc0863 (KatE), Bc0998 (YflT), Bc0999 (CsbD), Bc1002 (RsbV), Bc1003 (RsbW), Bc1004 (SigB), Bc1005 (bacterioferritin), Bc1010 (hypothetical), Bc1012 (hypothetical), Bc1154 (ferrochelatase), and Bc3132 (general stress protein) (Table 2). In line with these findings, significantly induced transcription was seen for genes that encode these general stress proteins (Supplementary Table S3B). Although the Bc1009 protein was not detected, the complementary transcriptional results showed that the *bc1009* gene was induced (Supplementary Table S3B).

Other newly identified members of the SigB-regulon mainly belong to the COG groups of cell motility, signal transduction mechanisms, transcription, amino acid transport, and metabolisms, or a group without assigned function (Fig. 3B, Tables 2 and 3). Representatives of the cell motility COG group included many flagella or chemotaxis proteins (FliM, FliN, FliG, YvzB, Bc1637, FlgE, PhnB, McpBH, YoaH, CheV, CheA, Bc0404, MotA, Bc0678, TlpA), and the signal transduction and transcription COG groups contained proteins that are related to sporulation (SigK, YndF, Spo0A, Bc4463)*.* Other strongly induced proteins included Bc0409 (carbamate kinase), Bc0566 (endonuclease/exonuclease phosphatase family protein), and Bc0666 (Immune inhibitor A precursor). Strikingly, a large number of transcriptional regulators were shown to be SigB-dependent, including SigK (sporulation sigma factor), Spo0A (sporulation initiation protein), CggR (central glycolytic regulator), ArsR family transcriptional regulator Bc0613, GntR family transcriptional regulator YvfI, PadR, YdgH, and the MerR family transcriptional regulator Bc3356 (Table 2). The most significantly induced protein—Bc0107 (YacN), a 2-C-methyl-D-erythritol 2,4-cyclodiphosphate synthase, has a putative role in lipid transport and metabolism. Together with the strongly induced protein Bc4345 that encodes a lipase, this suggests an additional role of SigB in the modulation of membrane lipid composition. Remarkably, a cluster of proteins (Bc5117—Bc5125) that is likely involved in the transport of nutrients, and Bc0423 (a non-ribosomal peptide synthetase that is involved in secondary metabolite production) also showed significant induction in wt cells and impaired production in Δ*sigB* cells after heat shock compared to before heat shock (Fig. 3B, Table 2).

**Table 3:** SigB-dependent downregulated proteins upon heat shock in *B. cereus* ATCC14579^**a**^

No	Locus Tag	Protein	Annotation	wt 42 °C/ Δ*sigB* 42 °C log2 FC	wt 42 °C/ Δ*bc1009* 42 °C log2 FC	COG
1	BC4320	RpsT	SSU ribosomal protein, translation	-6.2		J
2	BC1699	BC1699	ECF-type sigma factor negative effector	-4.9		S
3	BC2026	BC2026	Oligopeptide-binding protein oppA	-4.8		E
4	BC0450	YfkJ	Protein tyrosine phosphatase	-4.8		T
**5**	**BC3442**	**BC3442**	**hypothetical protein**	**-4.6**	**-1.2**	**R**
6	BC2675	BC2675	Acetyltransferase	-4.1		K
7	BC4168	BC4168	hypothetical protein	-4.1		S
**8**	**BC0066**	**YabR**	**S1-type RNA-binding domain**	**-3.5**	**-0.7**	**J**
9	BC3321	YfkO	NAD(P)H-dependent flavin reductase	-3.3		C
10	BC4935	YutM	Fe-S carrier protein, assembly of Fe-S clusters, DNA repair	-2.6		S
11	BC0544	BC0544	iron-sulfur cluster-binding protein	-2.5		C
12	BC4742	BC4742	ABC transporter permease protein	-2.5		V
13	BC0575	BC0575	hypothetical protein	-2.4		S
14	BC4619	BC4619	Protein with unknown function	-2.4		S
**15**	**BC2272**	**BC2272**	**Protein export protein prsA precursor**	**-2.4**	**-0.6**	**O**
**16**	**BC1650**	**FlgD**	**Basal-body rod modification protein flgD**	**-2.2**	**-1.7**	**N**
17	BC1970	BC1970	Protein with unknown function	-2.0		H
18	BC5166	BC5166	hypothetical protein	-1.9		S
19	BC5232	BC5232	Phosphoglycerol transferase	-1.9		M
**20**	**BC1593**	**YitH**	**Acetyltransferase**	**-1.9**	**-1.3**	**KR**
21	BC4102	YbaC	Alpha/beta hydrolase	-1.9		S
**22**	**BC2421**	**BC2421**	**hypothetical protein**	**-1.9**	**-0.7**	**S**
23	BC1137	AddB	ATP-dependent nuclease subunit B	-1.8		L
24	BC2084	BC2084	hypothetical Cytosolic Protein	-1.8		S
25	BC1548	BC1548	Endonuclease III	-1.8		L
**26**	**BC0949**	**BC0949**	**hypothetical Membrane Spanning Protein**	**-1.7**	**-1.7**	**EH**
27	BC3592	BC3592	Transcriptional regulator, TetR family	-1.7		K
**28**	**BC1638**	**BC1638**	**Flagellar hook-associated protein 2**	**-1.7**	**-1.5**	**N**
**29**	**BC5271**	**BC5271**	**UDP-N-acetylglucosamine 4-epimerase**	**-1.6**	**-1.4**	**MG**
30	BC0848	BC0848	Transcriptional regulator, AsnC family	-1.6		K
31	BC4448	BC4448	Protein with unknown function	-1.6		S
**32**	**BC0875**	**BC0875**	**hypothetical protein**	**-1.6**	**-1.2**	**S**
33	BC1991	BC1991	putative murein endopeptidase	-1.4		D
34	BC1520	YpiB	hypothetical Cytosolic Protein	-1.4		S
**35**	**BC0673**	**BC0673**	**Flavin-dependent dehydrogenase**	**-1.4**	**-1.1**	**C**
36	BC0489	BC0489	Glycosyltransferase involved in cell wall biogenesis	-1.4		M
37	BC2508	BC2508	Collagen adhesion protein	-1.4		M
**38**	**BC4652**	**YttP**	**Transcriptional regulator IcaR**	**-1.3**	**-0.8**	**K**
**39**	**BC3958**	**YktC**	**Myo-inositol-1(or 4)-monophosphatase**	**-1.3**	**-1.2**	**G**
40	BC3931	BC3931	hypothetical protein	-1.3		S
**41**	**BC5377**	**YwhC**	**Membrane metalloprotease**	**-1.3**	**-0.7**	**R**
**42**	**BC3609**	**BC3609**	**hypothetical protein**	**-1.3**	**-0.6**	**S**
**43**	**BC0984**	**BC0984**	**DNA-binding protein**	**-1.3**	**-1.2**	**S**
**44**	**BC2077**	**BC2077**	**YukE protein of unknown function**	**-1.3**	**-1.5**	**S**
**45**	**BC5274**	**YveM**	**UDP-N-acetylglucosamine 4,6-dehydratase**	**-1.3**	**-1.6**	**MG**
46	BC2060	BC2060	hydrolase (HAD superfamily)	-1.2		R
47	BC2926	BC2926	hypothetical protein	-1.2		L
**48**	**BC3437**	**BC3437**	**hypothetical Cytosolic Protein**	**-1.2**	**-1.6**	**S**
49	BC4150	YqiX	Arginine-binding protein	-1.2		ET
**50**	**BC0405**	**ArgR**	**Arginine repressor, argR**	**-1.2**	**-0.9**	**K**
**51**	**BC2298**	**BC2298**	**Transcriptional repressor**	**-1.1**	**-2.9**	**K**
52	BC0115	SecE	Protein translocase subunit SecE	-1.1		U
53	BC1545	YpmB	hypothetical protein	-1.1		S
**54**	**BC3953**	**YlaI**	**hypothetical protein**	**-1.1**	**-1.8**	**S**
**55**	**BC0235**	**YdaL**	**hypothetical protein**	**-1.1**	**-0.7**	**S**
56	BC4495	GerM	Germination protein germ	-1.1		R
57	BC4735	BC4735	hypothetical protein	-1.1		S
**58**	**BC4006**	**BC4006**	**hypothetical Cytosolic Protein**	**-1.1**	**-0.6**	**S**
59	BC5270	YvfC	Undecaprenyl-phosphate galactosephosphotransferase	-1.0		M
60	BC3791	YufN	Nucleoside-binding protein	-1.0		R
61	BC5126	BC5126	Transposase	-1.0		L
62	BC2248	YodQ	Acetylornithine deacetylase	-1.0		E
**63**	**BC0709**	**BC0709**	**Ferrous iron transport protein A**	**-1.0**	**-0.7**	**P**
**64**	**BC2578**	**BC2578**	**Phage protein**	**-1.0**	**-0.7**	**S**
**65**	**BC3398**	**BC3398**	**Serine transporter**	**-1.0**	**-0.8**	**E**
66	BC5401	YpmR	Lipase/Acylhydrolase with GDSL-like motif	-1.0		E
**67**	**BC4256**	**BC4256**	**Transcriptional regulator, ArsR family**	**-1.0**	**-1.0**	**K**
**68**	**BC5047**	**YvdC**	**IG hypothetical 16,995**	**-0.9**	**-1.3**	**R**
**69**	**BC0493**	**UgtP**	**1,2-diacylglycerol 3-glucosyltransferase**	**-0.9**	**-0.8**	**M**
70	BC3595	YvaA	Oxidoreductase	-0.9		R
71	BC0062	YabO	Heat shock protein 15	-0.9		J
72	BC4963	BC4963	hypothetical Cytosolic Protein	-0.9		S
73	BC0163	TruA	tRNA pseudouridine synthase A	-0.9		J
74	BC2770	GlcR	Transcriptional regulator, DeoR family	-0.9		KG
75	BC1641	FlgB	Flagellar basal-body rod protein flgB	-0.8		N
76	BC0563	BC0563	Biotin carboxyl carrier protein	-0.8		C
**77**	**BC4436**	**RpmA**	**LSU ribosomal protein L27P**	**-0.8**	**-1.3**	**J**
78	BC5327	BC5327	Stage II sporulation protein R	-0.8		S
79	BC0383	FhuD	Ferrichrome-binding protein	-0.8		P
80	BC1804	YbfQ	Rhodanese-related sulfurtransferases	-0.8		R
81	BC1517	AroB	3-dehydroquinate synthase	-0.7		E
**82**	**BC4830**	**BC4830**	**ABC transporter permease protein**	**-0.7**	**-0.7**	**V**
83	BC1365	YrdC	Isochorismatase	-0.7		Q
84	BC0851	YraB	Mercuric resistance operon regulatory protein	-0.7		K
85	BC4952	YutI	NifU protein	-0.7		O
**86**	**BC1901**	**BC1901**	**Phage protein**	**-0.7**	**-1.9**	**S**
87	BC3093	BC3093	hypothetical protein	-0.7		S
88	BC0873	YckJ	Cystine transport system permease protein	-0.7		E
**89**	**BC3209**	**BC3209**	**hypothetical Cytosolic Protein**	**-0.7**	**-1.4**	**S**
90	BC4789	LuxS	Autoinducer-2 production protein luxS / Ribosylhomocysteinase	-0.7		T
91	BC2054	GatA	Glutamyl-tRNA(Gln) amidotransferase subunit A	-0.7		J
**92**	**BC1984**	**BC1984**	**hypothetical protein**	**-0.7**	**-1.0**	**S**
93	BC0304	BC0304	FrnE protein	-0.7		Q
94	BC1188	YjbD	Arsenate reductase family protein	-0.6		P
95	BC0353	YerQ	hypothetical protein	-0.6		IR
96	BC0371	BC0371	Mandelate racemase/muconate lactonizing enzyme family protein	-0.6		MR
97	BC1648	BC1648	hypothetical Cytosolic Protein	-0.6		S
**98**	**BC4165**	**BkdR**	**Sigma-54-dependent transcriptional activator**	**-0.6**	**-1.0**	**KT**
99	BC2940	BC2940	Histidinol-phosphate aminotransferase	-0.6		E
100	BC1377	BC1377	hypothetical protein	-0.6		S
**101**	**BC2215**	**YfkC**	**Mechanosensitive ion channel**	**-0.6**	**-2.0**	**M**
**102**	**BC3380**	**BC3380**	**Quinone oxidoreductase**	**-0.6**	**-0.7**	**C**
103	BC4925	YumB	NADH dehydrogenase	-0.6		C
104	BC5312	AtpB	ATP synthase A chain	-0.6		C
**105**	**BC3548**	**YueD**	**Benzil reductase**	**-0.6**	**-2.9**	**IQR**
106	BC0449	BC0449	hypothetical protein	-0.6		S
107	BC4724	BC4724	Molybdenum cofactor biosynthesis protein A	-0.6		H
**108**	**BC2369**	**BC2369**	**Acetyltransferase**	**-0.6**	**-0.8**	**K**
109	BC1195	YjbI	Globin Family Protein	-0.6		R

^**a**^Bold: SigB and Bc1009-dependent-downregulated proteins under heat shock in *B. cereus*

Not Bold: SigB-dependent-induced proteins upon heat shock in *B. cereus*

log_2_ FC: log_2_ protein fold change values at either T20 or T40 timepoint

COG:Cluster of orthologous groups, see description in Fig. 3

Only SigB and Bc1009-dependent proteins are listed here

A total of 98 of the newly identified SigB-dependent proteins are also Bc1009-dependent, including flagella/chemotaxis proteins, transcriptional activator/repressors, ABC transporters, proteins involved in amino acid transport and metabolism, and cell wall/membrane/envelope biogenesis (Fig. 3B, and bold-highlighted in Table 2). Similarly, as reported above, the Bc5117- Bc5125 cluster and Bc0423 (non-ribosomal peptide synthetase) also showed reduced levels in Δ*bc1009* culture compared to wt cells. Bc0423 was also downregulated at the gene level in both mutants (complementing transcriptomic data are presented in Supplementary Table S3B. Several other proteins that showed significantly different levels in Δ*sigB* and Δ*bc1009* cultures versus wt do not belong to the COG groups mentioned above. For instance, YocJ (FMN-dependent NADH-azoreductase) was induced > 50-fold (log_2_ ~ 5.6) stronger in wt cultures compared to Δ*sigB* mutant, and > tenfold (log_2_ ~ 3.3) stronger in wt cultures compared to Δ*bc1009* mutant. Similar observations were made for other proteins with unknown functions (Bc4786, Bc5066, Bc3935 cytosolic protein, Bc5077, Bc0666 immune inhibitor A precursor) (see Table 2).

These results were supported by the transcriptomics data (Supplementary Table S3B), which showed differential transcription of genes encoding the reported motility proteins in Δ*sigB* and Δ*bc1009* cultures, pointing to a role of SigB and Bc1009 in the control of cell motility. Notably, despite the SigB-induced transcription of a group of phage genes, the corresponding encoded proteins were either not detected or not differentially produced in heat-stressed cells.

##### SigB-dependent and SigB/Bc1009- dependent downregulated proteins

On the other hand, 109 of 435 proteins that showed lower levels in wt cells after heat shock than before heat shock showed higher levels in the Δ*sigB* mutant, indicating that the expression of the encoding genes is likely indirectly regulated by SigB in wt cells (Fig. 2, Table 3). 40 of these 109 proteins also showed higher levels in the Δ*bc1009* mutant than in wt, suggesting that the encoding genes are directly or indirectly regulated by SigB and Bc1009 (Fig. 2, Table 3). The complete list of the SigB-dependent and SigB (and Bc1009)-dependent proteins that displayed lower levels after heat shock with their annotated functions is presented in Table 3. The log_2_ fold changes of the levels of these proteins are presented in the volcano plot (Fig. 3A), and their COG functions are shown in Fig. 3B. The top 20 SigB-dependent proteins with lower reductions of levels after heat shock in Δ*sigB* and Δ*bc1009* cells compared to wt are indicated by numbers in Fig. 3A on the left (marked in white), with five of these being SigB and Bc1009- dependent (marked in Grey in Fig. 3A) (see details in Table 3).

Many of the 109 proteins (Fig. 2, Table 3) that revealed SigB-dependent reductions in levels in wt cells after heat shock compared to control samples have undefined functions or fall into the COG group of proteins with transcription, cell wall biogenesis, and energy production and conversion functions (Fig. 3B, Table 3). For instance, the most prominently downregulated proteins in wt cells (~ 25 to 75 fold; log_2_ FC =  ~ 4.6—6.2) after heat shock vs. before heat shock include RpsT (SSU ribosomal protein, involved in translation, ribosomal structure, and biogenesis), Bc2026 (oligopeptide-binding protein OppA involved in amino acid transport and mechanism), Bc1699 (ECF-type sigma factor negative effector with unknown function), YkfJ (protein tyrosine phosphatase in signal transduction), and Bc3442 (hypothetical protein). Remarkably, several other proteins involved in transcription were significantly downregulated in wt cells but less so in Δ*sigB* and Δ*bc1009* cells, such as the ArgR arginine repressor, Sigma-54-dependent transcriptional activator GlcR regulator, TetR family regulator Bc3592, and ArsR family transcriptional regulator Bc4256 (Table 3).

For 40 of these proteins (Table 3, bold highlighted), the reduction in level after heat shock was not only dependent on SigB but also on Bc1009, and for many of them the function has not been defined yet. Those showing significant differential expression with known functions are mainly engaged in transcription, including YitH acetyltransferase, ArgR arginine repressor, Bc2298 transcriptional repressor, Bc4652 IcaR transcriptional regulator, Bc4256 ArsR family transcriptional regulator, BkdR sigma 54-dependent transcriptional activator, and Bc2369 acetyltransferase.

Transcriptional analysis generally supported proteomics data, except for a group of *nar* genes involved in anaerobic respiration; these showed SigB dependency in wt cells upon heat shock (Supplementary Table S3B), while no differential expression of corresponding proteins was observed (Table 3). Moreover, comparative proteomics and transcriptomics data of wt vs. Δ*bc1009* also showed an additional group of Bc1009-induced/downregulated proteins/genes that are not dependent on SigB. Several prominent Bc1009-induced proteins are transcriptional regulators and Bc1009-downregulated proteins are phage or transport proteins (Table S4A, Figure S3), but their exact roles in *B. cereus* in relation to heat-stress response are yet to be elucidated. As many of these proteins/genes have hypothetical functions and this study focussed on the SigB-mediated responses, these data are not further discussed here, but details are listed in Supplementary Table S4A (Figure S3) and S4B, respectively.

The results presented show more than 300 newly identified SigB-dependent proteins (Tables 2 and 3), and more than 100 of these require both SigB and Bc1009 for changes in their level in heat-stressed cells, indicating a significant extension of the *B. cereus* SigB regulon and a subregulon additionally requiring the Hpr-like phosphocarrier protein Bc1009. Most of these SigB and Bc1009-dependent proteins are involved in cell motility, signal transduction mechanisms, transcription, amino acid transport and metabolism, and cell wall biogenesis. Other proteins are responsible for DNA replication and repair, protein quality maintenance, and cell wall remodeling, suggesting a role of Bc1009 in adaptive heat stress response in *B. cereus* as well.

### Bc1009 and SigB contribute to survival of severe heat stress

**Fig. 4:**
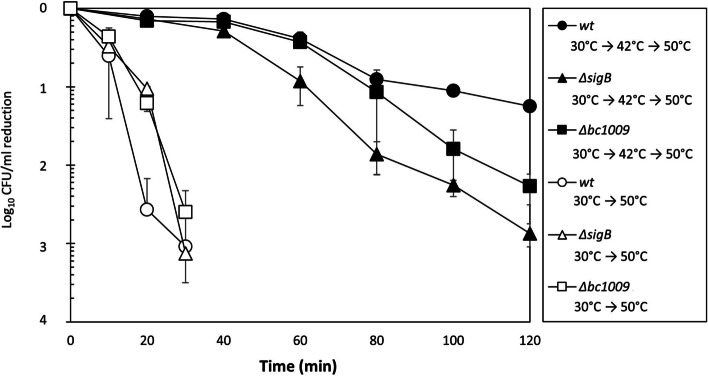
The relative survival of *B. cereus* wt, Δ*sigB,* and Δ*bc1009* mutants upon lethal heat exposure at 50°C. The relative survival at 50 °C of heat-preadapted cells (30 °C to 42 °C for 45 min) of *B. cereus* wt (filled circle), Δ*sigB* (filled triangle), and Δ*bc1009* cells (filled square) compared to cells that were not preadapted to heat (42 °C) (wt- open circle; Δ*sigB*- open triangle; Δ*bc1009*- open square) for 120 min. *N* = 4. *p* < 0.001 for time point at 100 min and 120 min when comparing wt and the two mutant strains. Error bars show the standard deviation of four biological replicates

To determine the impact of *bc1009* and *sigB* on survival of severe heat stress, the wt strain and isogenic mutants lacking Bc1009 or SigB were exposed to 50 °C, with or without pre-adaptation at 42 °C (Fig. 4). Pre-adaption of wt, Δ*sigB,* and Δ*bc1009* cultures resulted in a significantly higher survival rate at 50 °C than the non-pre-adapted control cells. The wt showed a 1.5 log_10_ reduction at 120 min. Although the adapted Δ*sigB*, Δ*bc1009,* and wt cultures showed similar survival during the first 40 min of exposure, the survival of the Δ*sigB* mutant then rapidly declined, resulting in approximately 2 log_10_ reductions after 120 min compared to the wt. The thermotolerance of adapted Δ*bc1009* cells was higher than that of Δ*sigB* cells. Survival rates were also similar to wt cells for the first 80 min, but a stronger decrease in survival was observed after 120 min at 50 °C with an approximate 1.3 log_10_ reduction compared to the wt. This points to a modest role of Bc1009 in activating heat stress defense, in line with its role in controlling the expression of a subset of SigB-dependent genes/proteins.

Exposure to 50 °C of cultures of wt, Δ*sigB* and Δ*bc1009* that were not pre-adapted at 42 °C showed rapid killing. After 20 min of exposure to this lethal heat stress, the wt showed a more prominent reduction than the two mutants however, a 3 log_10_ reduction was observed for all three strains after 30 min (Fig. 4).

### Impact of SigB and Bc1009 on the *B. cereus* protein profile under control conditions at 30°C

The impact of SigB and Bc1009 on the level of motility proteins after heat shock, and previous studies on the regulation of *B. cereus* motility at 30 °C by MogR and RpoN [52, 53], prompted us to perform an additional comparative omics analysis of non-stressed wt, Δ*sigB* and Δ*bc1009* cells at 30 °C.

**Fig. 5:**
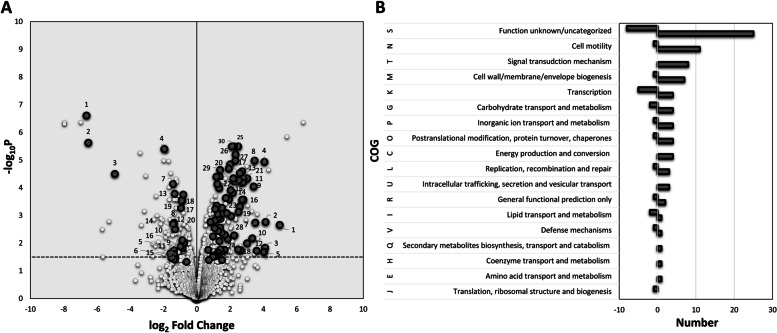
SigB-dependent induced and downregulated proteins for Δ*sigB* and Δ*bc1009* mutants compared to wt cells at 30°C and their cluster of orthologous group (COG). **A**- log_2_ fold change in protein expression. Positive and negative fold change values indicate induced and downregulated proteins, respectively. Grey symbols: induced/downregulated proteins that are SigB (and Bc1009)- dependent, i.e., differentially expressed proteins in Δ*sigB* and Δ*bc1009* mutants compared to wt cells at 30 °C; the numbers indicate the top 30 induced proteins (positive x-axis) or top 20 downregulated proteins (negative x-axis), with details listed in Table 4. White symbols: induced/downregulated proteins at 30 °C that are either only SigB-dependent or Bc1009-dependent, i.e., differentially expressed proteins in Δ*sigB* or Δ*bc1009* mutants compared to wt cells at 30 °C (see details in Supplementary Table S5A). The threshold of the *p*-value for each protein was < 0.05. *N* = 4. **B**- cluster of orthologous group (COG) function for SigB (and Bc1009)-induced and downregulated proteins. The number on the x-axis indicates the number of induced/downregulated proteins in respective COG groups. The underlying transcriptome data supporting this figure are presented in Supplementary Table S5B

In total, 96 proteins (Table 4) showed significant differences in level in both Δ*sigB* and Δ*bc1009* mutants compared to wt cells already at 30 °C, indicating that even their basal levels are dependent on SigB and Bc1009. This included 72 proteins with higher levels in the wt compared to the mutants and 24 with lower levels in the wt compared to the mutants (Fig. 5A, Table 4). The proteins displaying the largest SigB and Bc1009-dependent differences in levels are indicated with numbers in Fig. 5A on the right and left, respectively. The COG functions of these proteins are shown in Fig. 5B, and the complete list of differentially regulated proteins is presented in Table 4 (detailed in Supplementary Table S5A), with the complemented transcriptome data shown in Supplementary Table S5B. Interestingly, ~ 50% (41 of 72 induced and 12 of 24 downregulated) of the differentially expressed proteins that were detected at 30 °C were also differentially regulated in heat-stressed cells (bold highlighted in Table 4) and described above in the section Comparison of proteomic profiles of Δ*sigB* and Δ*bc1009* mutants to wt upon heat shock.

**Table 4:** Differentially regulated SigB and Bc1009-dependent proteins at 30°C in *B. cereus* ATCC14579^**a**^

No	Locus Tag	Protein	Annotation	SigB- dependent wt 30 °C / Δ*sigB* 30 °C log2 FC	Bc1009- dependent wt 30 °C / Δ*bc1009* 30 °C log2 FC	COG
1	BC5408	YfnB	2-haloalkanoic acid dehalogenase	5.0	3.9	R
**2**	**BC0423**	**SrfAAH**	**Non-ribosomal peptide synthetase (adenylation domain)**	**4.1**	**3.9**	**Q**
3	BC2294		hypothetical Cytosolic Protein	4.1	1.6	S
4	BC2724	OmdAH	LAAC/ Bacteriocin-protection, YdeI or OmpD-Associated	4.1	4.2	S
5	BC1718		DUF family protein of unknown function	4.0	4.9	S
6	BC3071	CutC	Copper homeostasis protein cutC	3.6	3.1	P
7	BC2752	YpeB	hypothetical Membrane Spanning Protein	3.5	3.5	S
**8**	**BC1303**	**YvfV**	**(S)-2-hydroxy-acid oxidase, iron-sulfur chain**	**3.5**	**3.0**	**C**
**9**	**BC5077**		**hypothetical protein**	**3.4**	**3.2**	**S**
10	BC4830		ABC transporter permease protein	3.3	1.0	V
11	BC0544		iron-sulfur cluster-binding protein	3.0	3.1	C
**12**	**BC1662**	**FliM**	**Flagellar motor switch protein fliM**	**3.0**	**2.9**	**N**
**13**	**BC5121**		**protein of unknown function**	**2.8**	**1.8**	**S**
**14**	**BC0576**	**McpBH**	**Methyl-accepting chemotaxis protein**	**2.8**	**1.4**	**NT**
**15**	**BC5243**		**protein with unknown function**	**2.8**	**2.6**	**S**
16	BC4496	RacE	Glutamate racemase	2.7	2.6	M
**17**	**BC3257**		**N-acetylmuramoyl-L-alanine amidase, Cell wall or membrane biogenesis**	**2.7**	**2.4**	**TM**
18	BC3698		Cell wall endopeptidase, family M23/M37	2.6	3.9	M
19	BC0232		hypothetical Membrane Spanning Protein	2.6	1.4	S
**20**	**BC0678**		**Methyl-accepting chemotaxis protein, signaling domain**	**2.6**	**2.0**	**NT**
**21**	**BC5123**		**homolog of lantibiotic biosynthesis dehydratase C-term**	**2.5**	**1.8**	**S**
22	BC4019		hypothetical protein	2.5	1.5	S
23	BC1202		Serine/threonine protein phosphatase	2.5	1.1	T
**24**	**BC1663**	**FliN**	**Flagellar motor switch protein fliN**	**2.5**	**2.8**	**NU**
**25**	**BC3406**		**Oxidoreductase**	**2.5**	**2.6**	**R**
**26**	**BC5118**		**ABC transporter ATP-binding protein**	**2.3**	**1.9**	**E**
27	BC1688	YmfQ	IG hypothetical 17,894	2.3	1.6	S
**28**	**BC4170**	**SpoOA**	**Stage sporulation protein A**	**2.2**	**1.3**	**TK**
**29**	**BC5125**		**Peptidase family M50**	**2.2**	**2.3**	**M**
30	BC5198	YviA/degV	DegV family fatty acid binding protein, phosphorylation of fatty acids	2.2	2.0	S
**31**	**BC5122**		**YcaO cyclodehydratase, ATP-ad Mg2 + -binding**	**2.2**	**1.9**	**S**
**32**	**BC5117**		**ABC transporter permease protein**	**2.2**	**1.7**	**S**
**33**	**BC5119**		**In operon with BC5120, which is a nitroreductase family protein**	**2.1**	**1.8**	**S**
**34**	**BC1857**	**SbcD**	**Exonuclease SbcD, DNA recombination and repair**	**2.1**	**2.8**	**L**
35	BC4512	MotB	Chemotaxis motB protein	2.0	0.7	N
**36**	**BC5103**	**YclP**	**Ferric anguibactin transport ATP-binding protein**	**2.0**	**1.7**	**P**
**37**	**BC1658**	**YvzB/ FliC**	**Flagellin**	**1.9**	**2.4**	**N**
**38**	**BC5141**	**CggR**	**Central glycolytic genes regulator**	**1.9**	**2.3**	**K**
39	BC1168	ClpB	ClpB protein	1.9	1.5	O
**40**	**BC1651**	**FlgE**	**Flagellar hook protein flgE**	**1.8**	**1.8**	**N**
41	BC5128	SmpB	SsrA-binding protein, required for rescue of stalled ribosomes	1.7	1.7	O
42	BC4703	YtzE	Transcriptional regulator, DeoR family	1.7	1.1	KG
**43**	**BC5034**	**YoaH**	**Methyl-accepting chemotaxis protein**	**1.7**	**1.1**	**NT**
44	BC4403	YrvE	Single-stranded-DNA-specific exonuclease recJ	1.5	1.3	LS
**45**	**BC5410**	**YocJ**	**FMN-dependent NADH-azoreductase**	**1.5**	**1.1**	**I**
**46**	**BC5120**		**hypothetical Cytosolic Protein**	**1.5**	**1.3**	**S**
47	BC1850		Transcriptional regulator	1.5	1.0	K
48	BC1660	YjbJ	Soluble lytic murein transglycosylase	1.4	1.8	M
49	BC4984		ABC transporter substrate-binding protein	1.4	1.1	P
**50**	**BC5255**	**YknX**	**periplasmic component of efflux system**	**1.4**	**1.1**	**M**
**51**	**BC1654**	**CheV**	**Chemotaxis protein cheV**	**1.4**	**1.0**	**NT**
**52**	**BC2285**	**MmgD**	**2-methylcitrate synthase**	**1.4**	**1.3**	**C**
**53**	**BC2464**		**S-layer protein / Peptidoglycan endo-beta-N-acetylglucosaminidase**	**1.4**	**2.0**	**G**
54	BC1639	Flis	Flagellar protein fliS	1.3	1.0	NUO
**55**	**BC0888**	**CwlH**	**N-acetylmuramoyl-L-alanine amidase**	**1.3**	**1.2**	**M**
56	BC3947	PycA	Pyruvate carboxylase	1.3	1.0	C
57	BC3081	YhjR	hypothetical protein	1.3	1.2	S
58	BC0896		S-layer protein / Peptidoglycan endo-beta-N-acetylglucosaminidase	1.2	0.9	G
**59**	**BC1657**		**hypothetical protein**	**1.2**	**1.5**	**S**
**60**	**BC5436**		**Peptide methionine sulfoxide reductase**	**1.2**	**0.9**	**O**
**61**	**BC1659**		**hypothetical protein**	**1.2**	**1.0**	**S**
**62**	**BC0404**	**TarH**	**Methyl-accepting chemotaxis protein (motility, Signal transduction)**	**1.2**	**1.6**	**NT**
**63**	**BC2431**	**PhnB**	**PhnB protein**	**1.1**	**0.6**	**S**
**64**	**BC0597**	**YueK**	**Nicotinate phosphoribosyltransferase**	**1.1**	**1.5**	**H**
65	BC1157		Alpha-amylase	1.1	0.8	G
66	BC1653		hypothetical protein	1.1	0.7	S
**67**	**BC4178**		**Exodeoxyribonuclease VII small subunit**	**1.0**	**0.7**	**L**
68	BC1726		hypothetical Membrane Spanning Protein	1.0	1.8	S
**69**	**BC0675**		**hypothetical protein**	**1.0**	**2.0**	**S**
70	BC5189	SecA	Protein translocase subunit SecA	0.8	1.1	U
**71**	**BC5241**	**YvbJ**	**IG hypothetical 16,680**	**0.7**	**1.3**	**S**
**72**	**BC4791**	**YtiB**	**Carbonic anhydrase**	**0.7**	**1.3**	**P**
1	BC0385		Thioredoxin reductase, posttranslational modification, protein turnover, chaperones	-6.6	-5.8	O
**2**	**BC0405**	**ArgR**	**Arginine repressor, argR**	**-6.5**	**-6.5**	**K**
3	BC2355		hypothetical protein	-4.9	-3.2	S
**4**	**BC2077**		**YukE protein of unknown function**	**-2.0**	**-2.0**	**S**
5	BC3587		Transcriptional regulator, LytR family	-1.6	-0.8	K
6	BC1363	LrpC	Leucine-responsive regulatory protein	-1.5	-0.9	K
7	BC3728		hypothetical protein	-1.4	-1.4	L
**8**	**BC1520**	**YpiB**	**hypothetical Cytosolic Protein**	**-1.4**	**-1.1**	**S**
9	BC5341	AcdA	Acyl-CoA dehydrogenase, short-chain specific	-1.4	-1.5	I
10	BC1359	YhcH	Bacitracin transport ATP-binding protein bcrA	-1.4	-0.6	V
11	BC5101		Perfringolysin O precursor	-1.4	-2.4	S
**12**	**BC3281**		**hypothetical protein**	**-1.4**	**-0.9**	**S**
**13**	**BC1638**	**Flg**	**Flagellar hook-associated protein 2**	**-1.3**	**-1.5**	**N**
14	BC5002	YusJ	Acyl-CoA dehydrogenase	-1.3	-0.9	I
15	BC1914		Phage protein	-1.3	-0.7	K
16	BC0366		hypothetical protein	-1.0	-0.7	S
**17**	**BC4256**		**Transcriptional regulator, ArsR family**	**-1.0**	**-0.8**	**K**
**18**	**BC5422**		**hypothetical protein**	**-0.9**	**-0.8**	**S**
**19**	**BC5274**	**YveM**	**UDP-N-acetylglucosamine 4,6-dehydratase**	**-0.9**	**-1.2**	**MG**
20	BC3731	YvgY	COP associated protein	-0.9	-0.7	P
21	BC0679		Cell wall-binding protein	-0.9	-1.3	S
22	BC0858		Modulator of drug activity B	-0.8	-0.7	R
23	BC0157	RpsK	SSU ribosomal protein S11P	-0.8	-0.8	J
24	BC5335	FbaA	Fructose-bisphosphate aldolase	-0.6	-0.7	G

COG:Cluster of orthologous groups, see description in Fig. 5

^**a**^Bold: SigB and Bc1009-dependent-proteins that were also differentially regulated upon heat shock in *B. cereus*

Not Bold: SigB and Bc1009-dependent proteins uniquely regulated at 30 °C in wt cells versus Δ*sigB* and Δ*bc1009* mutants

log_2_ FC: Protein fold change values at T0 time point

Only SigB and Bc1009-dependent proteins are listed here

#### SigB and Bc1009-dependently induced proteins at 30°C

SigB and Bc1009-dependent induced proteins at 30 °C include flagella and chemotaxis proteins, transcriptional regulators like Spo0A and CggR, the Bc5117- Bc5125 cluster (conceivably involved in transporting nutrients), and Bc0423 (Non-ribosomal peptide synthetase) (Table 4). Next to proteins that were also differentially expressed under heat shock (described above in the section  Impact of SigB and Bc1009 on B. cereus protein profiles after heat shock), an additional group of proteins involved in carbohydrate/ion transport and metabolisms was present at lower levels in Δ*sigB* and Δ*bc1009* cells compared to wt cells at 30 °C (Fig. 5B, Table 4). These were YtzE, Bc2464, and Bc0896- Peptidoglycan endo-beta-N-acetylglucosaminidase, Bc1157- alpha-amylase, CutC- copper homeostasis protein, YclP-ferric transport ATP binding protein, Bc4984 ABC transporter, YtiB carbonic anhydrase, and YvgY copper chaperon associated protein. The remaining uniquely SigB and Bc1009-induced proteins at 30 °C mainly have undefined functions or are additional proteins found in the same COG groups described above, including motility (Table 4). Proteomics results were supported by transcriptional data (Supplementary Table S5B).

#### SigB and Bc1009-dependently downregulated proteins at 30°C

An additional 24 proteins (Table 4) were present at lower levels in wt cells compared to Δ*sigB* and Δ*bc1009* mutants at 30 °C, of which 12 also displayed lower levels in wt cells upon heat shock, and 12 were uniquely regulated at 30 °C (Table 4). Four SigB and Bc1009-dependent downregulated proteins have putative roles in transcription regulation, including LytR family transcriptional regulator (Bc3587), leucine-responsive regulatory protein LrpC (Bc1363), ArsR family transcriptional regulator (Bc4256) and arginine repressor ArgR (Bc0405), of which the latter two were also upregulated in heat-stressed Δ*sigB* and Δ*bc1009* mutants compared to wt (Impact of SigB and Bc1009 on B. cereus protein profiles after heat shock section). Remarkably, the arginine repressor ArgR (Bc0405) and Bc0385 thioredoxin reductase were both present at 100-fold lower levels (log_2_ fold change ~ 6) in wt cells compared to Δ*sigB* and Δ*bc1009* mutants (Fig. 5, Table 4). However, genes that encode these proteins did not show differential expression (Supplementary Table S5B), suggesting regulation at the post-transcriptional level.

Taken together, this section provides evidence that SigB may be active already during control conditions at 30 °C, showing its alternative role in cellular functions other than the SigB GSR, potentially via the putative Hpr-like protein, Bc1009.

#### Δ*bc1009* and Δ*sigB* mutants both show a defective motility phenotype

**Fig. 6:**
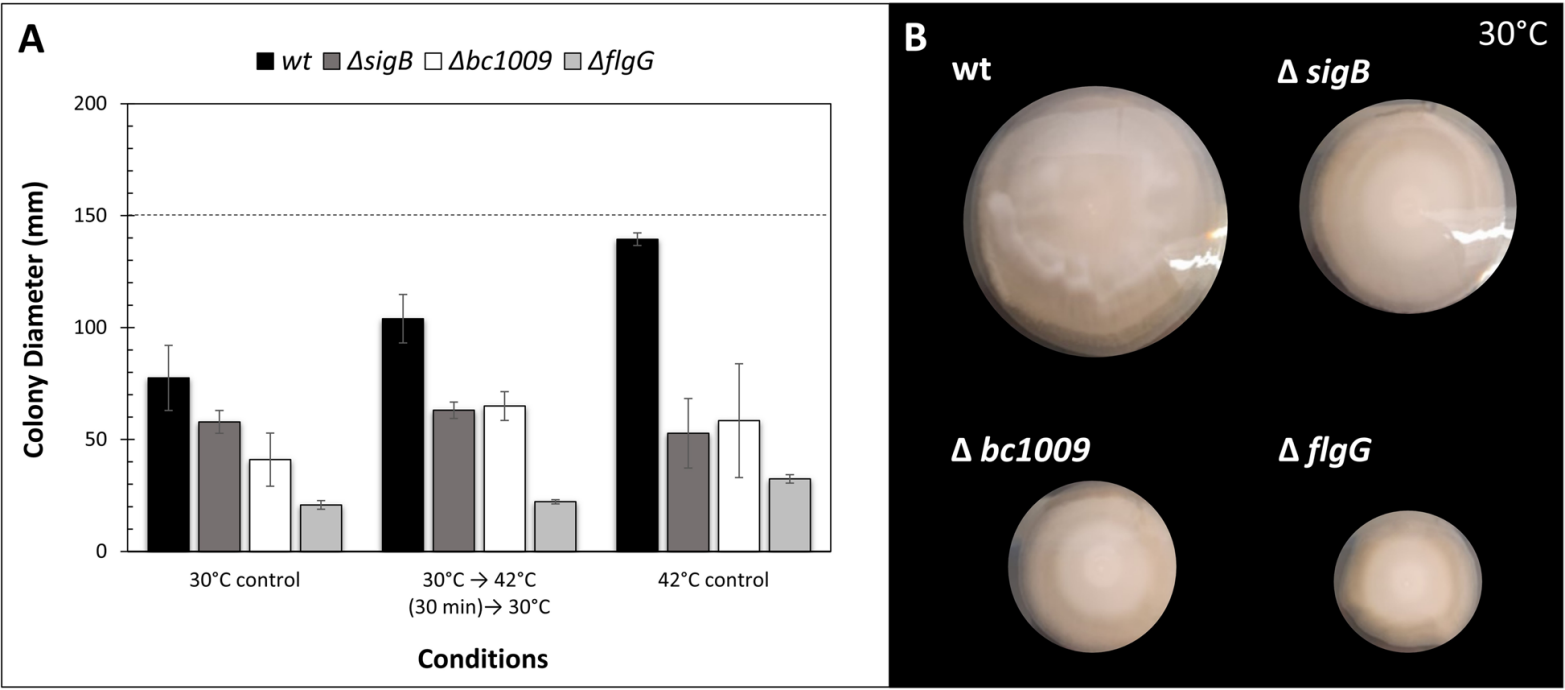
Motility phenotype of wt, Δ*sigB,* and Δ*bc1009* mutants. **A**- the motility of wt, Δ*sigB,* and Δ*bc1009* was compared on Brain Heart Infusion (BHI) agar with 0.25% agar and indicated by the colony diameter (mm) formed on the agar after 24 h incubation. Black bar: wt; Grey bar: Δ*sigB* mutant; white bar: Δ*bc1009* mutant; light grey bar: Δ*flgG* mutant (negative control without flagella). The motility of all cells was tested under three different conditions, 1) at 30 °C for 24 h; 2) upon heat shock from 30 °C to 42 °C for 30 min, and back to 30 °C for 24 h; and 3) at 42 °C for 24 h. The dotted line shows the maximum plate size. **B**- Colony of wt, Δ*sigB,* Δ*bc1009,* and Δ*flgG* cells on 0.25% BHI agar at 30 °C after 24 h. Error bars indicates the standard deviation of colony diameters of four biological replicates

Based on the observation that many motility/chemotaxis proteins were present at higher levels in both non-stressed and heat-stressed wt cells compared to non-stressed and heat-stressed Δ*sigB* and Δ*bc1009* mutants, we compared the motility of Δ*sigB* and Δ*bc1009* mutants with that of wt cells on BHI agar plates with a low agar percentage (0.25%) under three conditions: 1) at 30 °C (isothermal); 2) at 30 °C, following a heat-shock at 42 °C for 30 min; and 3) at 42 °C (isothermal). A mutant unable to produce flagella (Δ*flgG*) was used as a negative control. Results that are presented in Fig. 6 show that at 30 °C, the colony diameter of Δ*flgG* mutant was lowest, followed by that of Δ*bc1009*, then Δ*sigB*, and with the wt showing the highest motility (Fig. 6 left and right; observed phenotypes). Following a mild heat shock for 30 min at 42 °C and subsequent incubation at 30 °C, again, the colony diameter of Δ*flgG* mutant was lowest, with both Δ*sigB* and Δ*bc1009* mutants displaying intermediate levels of motility, with the wt displaying the highest motility. Similarly, incubation at isothermal 42 °C showed the lowest and highest motility of Δ*flgG* mutant and wt cells, respectively, while Δ*sigB* and Δ*bc1009* cells showed comparable intermediate motility. These results show that SigB-induced motility in non-heat-stressed and heat-stressed cells depends on Bc1009.

## Discussion

This study investigated the potential of *bc1009* that encodes the Hpr-like phosphocarrier protein Bc1009 in regulating SigB and/or modulating the expression of SigB regulon members. The Bc1009 protein is a *B.* *cereus* specific protein that is not found other *Bacillacea* family members, such as *B. subtilis, B. licheniformis* and *Listeria monocytogenes* (data not shown)*.* Comparative gene expression analysis of wt, *sigB*, and *bc1009* mutants ruled out a possible role of Bc1009 in regulating SigB expression, as its absence did not affect the expression of *sigB* and other SigB gene cluster members. Further proteomics studies of *B. cereus* wt, *sigB* and *bc1009* mutants provided novel insights into the SigB regulon and a role for Bc1009 in controlling the expression of a subset of genes/proteins in non-heat-stressed cells at 30 °C and heat-stressed cells at 42 °C.

Proteomics analyses backed up by transcriptomics data in this study revealed at least 284 SigB-controlled proteins upon heat shock in *B. cereus* (175 significantly induced/upregulated and 109 downregulated) (See Tables 2 and 3), including most SigB regulon members that were previously reported by De Been et al. [12]. In the current study, we excluded *rsbK* and *rsbM* from the SigB regulon list because the expression of *rsbK*, with *rsbM* in the same operon, was shown to be independent of SigB (Fig. 1)*.* Notably, a total of 96 SigB-dependent proteins (72 induced; 24 downregulated in Tables 2 and 3) were identified at 30 °C for non-heat-stressed cells (Table 4). Of these, 51/96 (53%) proteins overlapped with the results of SigB-dependent proteins that were identified upon heat shock, indicating that an additional 45 members were significantly differentially expressed at 30 °C (i.e., differentially expressed proteins in Δ*sigB* mutant compared to wt), potentially expanding the SigB regulon members to more than 300 members in *B. cereus.* The presumed size of the SigB regulon in *B. cereus* is thereby comparable with the size of the SigB regulons of *B. subtilis* [8, 24, 54] and *L. monocytogenes* [25, 26]. At least 300 SigB regulon members have been described for the latter two species.

The *B. subtilis* SigB regulon contained SigB-dependent members (i.e., genes/proteins) that are either directly or indirectly controlled by SigB, based on the presence or absence of SigB promoter binding motif (PBM), respectively [17, 55, 56]. SigB regulon members indirectly controlled by SigB may occur via a cascade effect through a direct SigB-controlled regulator/protein. In this study, we provided evidence that ~ 70% of the newly identified SigB regulon members in *B. cereus* were regulated via the direct SigB-controlled member, Hpr-like phosphocarrier protein Bc1009. In line with this, all genes/gene clusters encoding newly identified proteins do not contain SigB PBMs (data not shown). The full set of directly and indirectly regulated SigB genes and gene clusters should be referred to as the SigB modulon [57, 58]. Several of these proteins appeared to contribute to the heat stress response, as proteins with putative functions in heat stress resistance (e.g., DNA recombination and repair, cell wall remodeling, and protein quality maintenance) were present at lower levels in Δ*bc1009* and Δ*sigB* mutants compared with wt upon heat shock. In line with these findings, mild heat-induced stress resistance of the Δ*bc1009* mutant was lower than that of wt cells but higher than the Δ*sigB* mutant when lethal heat stress was imposed.

A group of sporulation-related proteins (with COG functions of transcription/signal transduction) was also found to be inducible via SigB after heat shock (SigK sporulation sigma factor, Bc4463, Spo0A, YndF, Bc3576), with the expression of the latter three being dependent on Bc1009. Previously, a role of SigB in *B. cereus* sporulation has been reported [59]. In addition, studies in *B. subtilis* have also shown that SigB impairs sporulation initiation by inactivating the sporulation master regulator Spo0A via the Spo0E phosphatase [27, 28].

**Fig. 7:**
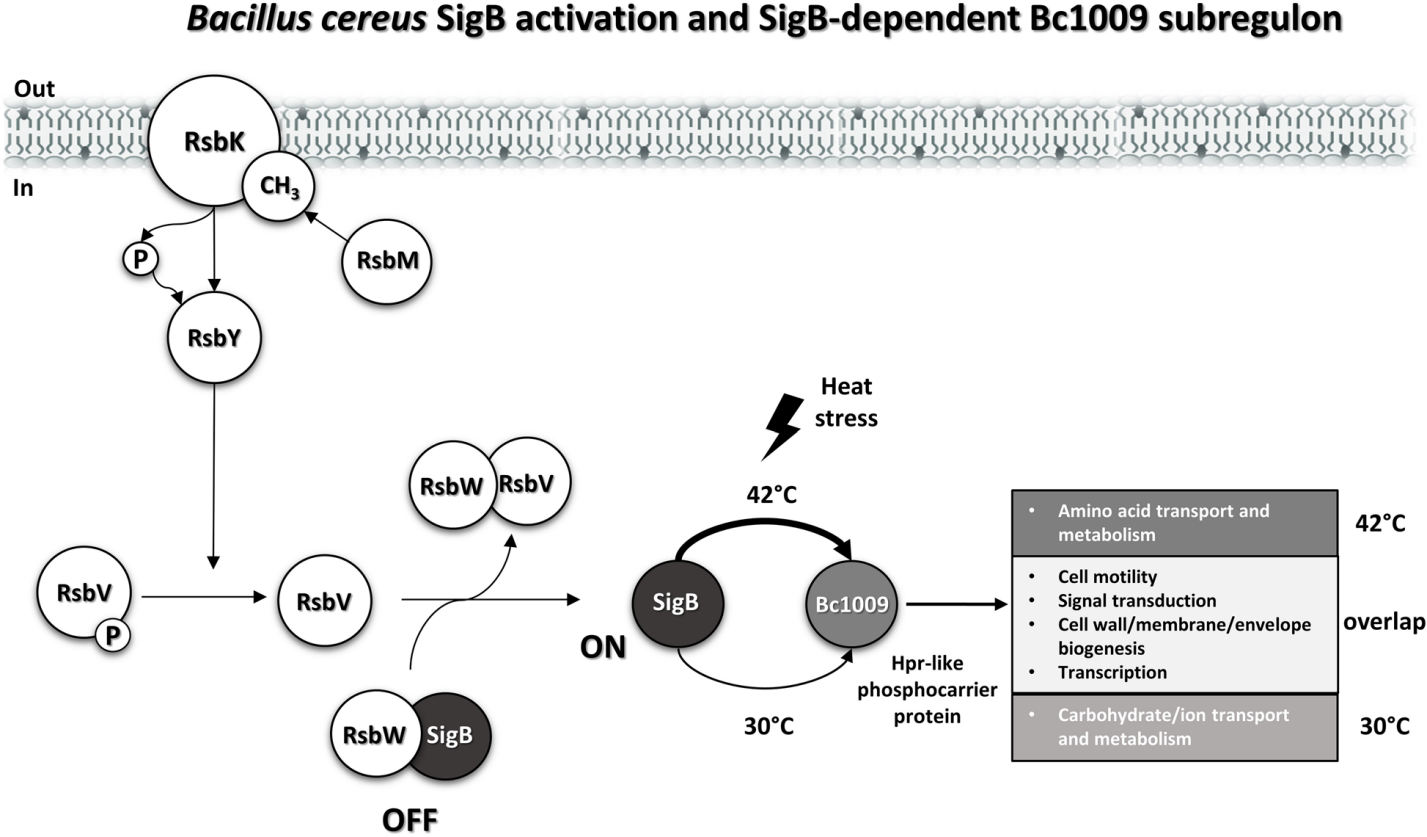
Activation model of *Bacillus cereus* SigB and the SigB-dependent Bc1009 subregulon. The RsbKY two-component system mediates the SigB general stress response in *B. cereus.* Under heat shock (temperature shift from 30 °C to 42 °C), SigB is induced (bold arrow) via the RsbKY signaling cascade and directly regulates the Hpr-like phosphocarrier protein Bc1009 to mediate amino acid transport and metabolism, cell motility, signal transduction, cell wall/membrane/envelope biogenesis and transcription. At 30 °C, the baseline level of SigB also appears to regulate Bc1009 (non-bold arrow) to mediate similar functions as described for the heat shock condition, except for amino acid transport and metabolism (which switch to carbohydrate/ion transport and metabolism at 30 °C instead, indicated in dark grey and light grey box, respectively). Upon SigB activation, other SigB regulon members not controlled by Bc1009 are also activated (see main text, Tables 2 and 3, and Supplementary Tables S5A and B). White color circles: known SigB signalling pathway as described in the literature [5, 12, 15, 16], Grey and Black circles and boxes: new insight obtained in the current study (see text for details)

Other SigB and Bc1009-dependent proteins have putative functions in cell motility, signal transduction mechanism, transcription, and cell wall/membrane/envelope biogenesis. Approximately 40% of these proteins were differentially regulated in non-heat-stressed control cells, suggesting that the baseline level of SigB at 30 °C without further induction already contributes to the regulation of cellular functions (Fig. 7). The significant induction of a large group of motility/chemotaxis proteins and the observed defect in motility phenotypes in Δ*sigB* and Δ*bc1009* mutants strongly suggests that SigB positively regulates motility in *B. cereus*. Based on this evidence, SigB can be added to the list of regulators, including MogR [53] and RpoN [52], that play a role in the control of flagella synthesis and motility/chemotaxis in *B. cereus*. As *B. cereus* wt, Δ*sigB,* and Δ*bc1009* mutants contained flagella (Supplementary Figure S2), higher motility of wt than the two mutants may be linked to the increased levels of motor switch proteins (FliN, FliM, and FliG) and/or chemotaxis proteins (Bc0422, TlpA, Bc0678, MotA, MotB, TarH, CheA, CheV, McpB/H, and YoaH). In *B. subtilis*, indirect roles of SigB in surfactin production and swimming and swarming activity have been reported [29, 30]. Interestingly, Bc0423, a homolog of surfactin synthetase, was substantially downregulated in SigB and Bc1009 mutants compared to wt in non-heat-stressed cells at 30 °C and heat-stressed cells at 42 °C. However, further studies are required to confirm the role of Bc0423 in *B. cereus* motility in the tested conditions.

Our data also showed that proteins with putative functions in the modulation of membrane composition were under the control of SigB. This is in line with an earlier report on the putative functions of augmented SigB regulon members predicted for clade A *B. cereus *sensu lato group, including *B. cereus* ATCC14579 [23]. In addition, the expression of the *sinI* gene (antagonist of *sinR,* a regulator in biofilm formation) was significantly reduced in the *sigB* mutant but not in the *bc1009* mutant, implying that SigB may play a role in biofilm formation in *B. cereus,* in line with observations showing that the Δ*sigB* mutant of *B. cereus* 905 forms a weaker biofilm than the wt [60]. Moreover, SigB has been reported to regulate biofilm aging and cell dispersal in *B. subtilis* (reviewed in Rodriguez Ayala et al. [8].

In non-heat-stressed cells of *B. cereus,* proteins that showed SigB-dependent differential expression were involved in carbohydrate/ion transport and metabolisms, while upon a heat shock from 30 °C to 42 °C, a switch was observed to the expression of proteins involved in amino acid transport and metabolisms (Fig. 7). This may indicate a rearrangement of cell resources to cope with heat stress in *B. cereus.* It is conceivable that amino acids are needed to maintain protein quality homeostasis under heat stress (e.g., as building blocks to aid protein repair/degradation). To the best of our knowledge, no known amino acid metabolism regulator has been reported to be SigB-controlled in *B. cereus* [23, 61] or *B. subtilis* [62]*.* However, the global regulator of branched-chain amino acid limitation in *Bacillus* species, CodY, is found to be SigB-dependent upon heat shock in *B. licheniformis* [63]. Nonetheless, based on information obtained in this study, SigB does not regulate CodY in *B. cereus,* or *B. subtilis* [62]*.*

*B. cereus* SigB (and Bc1009) dependent transcriptional regulators such as ArgR (arginine biosynthesis transcriptional regulator) may also play a role in metabolic shifts. Van Schaik et al. [10] previously showed that *B. cereus* failed to utilize a variety of nitrogen sources, including L-arginine, in the absence of SigB, suggesting that SigB may be linked to the control of nitrogen metabolism. Furthermore, SigB interaction with ArgR has also been described in *L. monocytogenes,* in which SigB is repressed by ArgR when arginine is absent and de-repressed by ArgR when arginine is present, thus forming feedback regulation [64].

SigB may also be indirectly engaged in controlling virulence factors in *B. cereus.* The expression of two genes (*bc3103* and *bc3104*) that encode Hemolysin BL lytic component L1 (Hbl-L1) and L2 (Hbl-L2), and two genes (*bc1809* and *bc1810*) that encode non-hemolytic enterotoxin component NheA and NheB, was de-repressed in the Δ*sigB* mutant after heat shock, and the expression of the *cytK* gene that encodes the cytotoxin CytK was induced in both Δ*sigB* and Δ*bc1009* mutants under the non-heat-stressed condition in this study. Notably, in *B. anthracis,* a Δ*sigB* mutant was found to be less virulent in the mouse model [65, 66], whereas in *B. thurigiensis,* SigB was required for its pathogenicity towards insect larvae and is required for adaptation to the insect gut environment [67]. We also found that Bc0385 (thioredoxin reductase) was dependent on SigB (and Bc1009). This protein is crucial for resistance against various disinfectant treatments in *B. cereus* [68], suggesting further involvement of SigB in controlling other stresses. However, the impact of SigB on emetic and diarrheal toxin production and virulence in *B. cereus* remains to be elucidated.

It is known that the phosphotransferase system (PTS) components in *Bacillus* regulate carbon utilization and carbohydrate uptake/transport in response to metabolic/environmental challenges [69]. This system links with the control of chemotaxis and cell motility, which supports the ability to scavenge additional carbon and/or nitrogen sources in the environment [70]. Such regulation is controlled via phosphorylation of target proteins, including transcriptional regulators, signal transduction proteins, transporters, and catabolic enzymes [69, 70]. Our results provide evidence for an additional level of control exerted by Hpr-like phosphocarrier protein Bc1009, following the stress-induced RsbKY-dependent activation of SigB in *B. cereus*. Such indirect control of SigB via Bc1009 may be exerted via the phosphogroup transfer that leads to the modulation of other two-component systems or transcriptional regulators, e.g., those involved in chemotaxis/motility and PTS-type carbohydrate transport and utilization. Despite the significant increase in putative SigB regulon members, the number of genes/proteins that SigB directly controls and that contain a SigB PBM is low, i.e., around 30 of 300, an approximate 10%. The stark contrast between the current and previously reported size of the *B. cereus* SigB regulon can be explained by the advanced proteomics approach used in the current study and the heat shock conditions used, which constituted a prolonged exposure time of 20–40 min to mild heat versus 10 min in the previous studies [10, 12, 71]. The established relatively small SigB direct regulon may be related to the use of different stress sensing systems, i.e., RsbKY in *B. cereus* versus RsbRST and/or RsbQP in *B. subtilis* and *L. monocytogenes.* The latter two species have at least 100 SigB direct regulon members with a SigB PBM [19, 24, 26].

## Conclusion

This study revealed novel SigB regulon members for *B. cereus* and provided evidence that expression of a SigB subregulon is controlled by Hpr-like phosphocarrier protein Bc1009. These subregulon members contribute to heat stress resistance and cell motility and have putative functions in signal transduction, cell wall/membrane/envelope biogenesis, transcription, amino acid/carbohydrate/ion transport and metabolism (Fig. 7)*.* Further exploration is required to investigate the possible roles of RsbKY in SigB-dependent or independent activation of Bc1009 upon exposure to other stresses and its impact on fitness and survival efficacy. In addition, identifying Bc1009 phosphotransfer-based interaction partners may shed light on other cellular regulatory networks.

## Supplementary Information

**Additional file 1:** **Supplementary Figure S1.** Heat regulon genes/proteins in *Bacillus cereus* ATCC14579. **Supplementary Figure S2.** Flagella quick staining of wt cells (A), Δ*sigB *cells (B) and Δ*bc1009* cells (C). Flagella indicated with yellow arrow. **Supplementary Figure S3**. BC1009- dependent proteins after heat shock. **Supplementary Table S1.** Oligonucleotides used in this study. **Supplementary Table S2A.** Induced and downregulated proteins upon heat shock (30°C to 42°C) in *Bacillus cereus* ATCC14579 wt. **Supplementary Table S2B.** Induced and downregulated genes upon heat shock (30°C to 42°C) in *Bacillus cereus* ATCC14579 wt. **Supplementary Table S3B.** SigB-dependent induced and downregulated genes upon heat shock (30°C to 42°C) in *Bacillus cereus* wt cells versus Δ*sigB* and Δ*bc1009* mutants. **Supplementary Table S4A.** Bc1009-dependent proteins in *Bacillus cereus *upon heat shock (30°C to 42°C) in wt cells versus Δ*bc1009* mutant. **Supplementary Table S4B.** Bc1009-dependent genes in *Bacillus cereus* upon heat shock (30°C to 42°C) in wt cells versus Δ*bc1009* mutant. **Supplementary Table S5A.** Differentially regulated SigB- dependent or SigB and Bc1009-dependent proteins in non-heat-stressed condition at 30°C in *B. cereu*s ATCC14579 wt cells versus Δ*sigB* and Δ*bc1009* mutants. **Supplementary Table S5B.** Differentially regulated SigB- dependent or SigB and Bc1009-dependent genes in non-heat-stressed condition at 30°C in *B. cereus* ATCC14579 wt cells versus Δ*sigB* and Δ*bc1009* mutants.

## Data Availability

All data generated or analysed during this study are included in this published article (and its supplementary information files). The raw mass spectrometry data and ISOQuant result file have been deposited at the MassIVE database (https://massive.ucsd.edu) under the project ID and accession number of MSV000090315. The raw DNA array data has been deposited to the Gene Expression Omnibus (GEO) database (https://www.ncbi.nlm.nih.gov/geo/) with the accession number of GSE213958.

## Abbreviations

GSR: General stress response
SigB: Sigma factor B
Rsb: Regulator of Sigma factor B
RT: Room temperature
PBM: Promoter binding motif
PTS: Phosphotransferase system
