# Supplementary figures and images for: SigB modulates expression of novel SigB regulon members via Bc1009 in non-stressed and heat-stressed cells revealing its alternative roles in Bacillus cereus
Source: BMC Microbiol. 2023 Feb 10;23:37. doi: 10.1186/s12866-023-02783-3 (PMC9912610; doi:10.1186/s12866-023-02783-3)

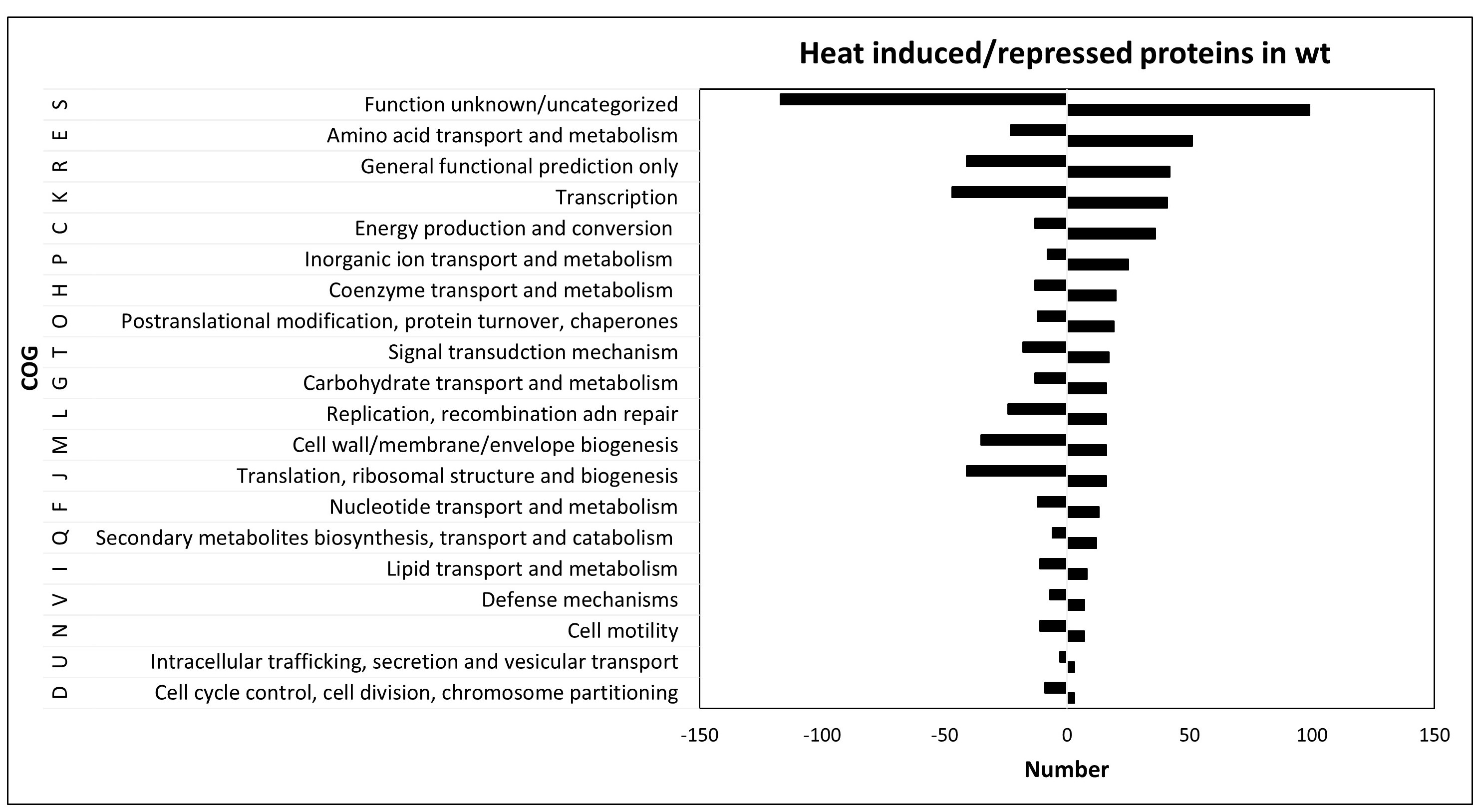

Supplement: Supplementary file 1 — Additional file 1: Supplementary Figure S1. Heat regulon genes/proteins in Bacillus cereus ATCC14579. Supplementary Figure S2. Flagella quick staining of wt cells (A), ΔsigB cells (B) and Δbc1009 cells (C). Flagella indicated with yellow arrow. Supplementary Figure S3. BC1009- dependent proteins after heat shock. Supplementary Table S1. Oligonucleotides used in this study. Supplementary Table S2A. Induced and downregulated proteins upon heat shock (30°C to 42°C) in Bacillus cereus ATCC14579 wt. Supplementary Table S2B. Induced and downregulated genes upon heat shock (30°C to 42°C) in Bacillus cereus ATCC14579 wt. Supplementary Table S3B. SigB-dependent induced and downregulated genes upon heat shock (30°C to 42°C) in Bacillus cereus wt cells versus ΔsigB and Δbc1009 mutants. Supplementary Table S4A. Bc1009-dependent proteins in Bacillus cereus upon heat shock (30°C to 42°C) in wt cells versus Δbc1009 mutant. Supplementary Table S4B. Bc1009-dependent genes in Bacillus cereus upon heat shock (30°C to 42°C) in wt cells versus Δbc1009 mutant. Supplementary Table S5A. Differentially regulated SigB- dependent or SigB and Bc1009-dependent proteins in non-heat-stressed condition at 30°C in B. cereus ATCC14579 wt cells versus ΔsigB and Δbc1009 mutants. Supplementary Table S5B. Differentially regulated SigB- dependent or SigB and Bc1009-dependent genes in non-heat-stressed condition at 30°C in B. cereus ATCC14579 wt cells versus ΔsigB and Δbc1009 mutants. [file 12866_2023_2783_MOESM1_ESM.zip › Figure S1 heat induced or and repressed proteins in wt.jpg]

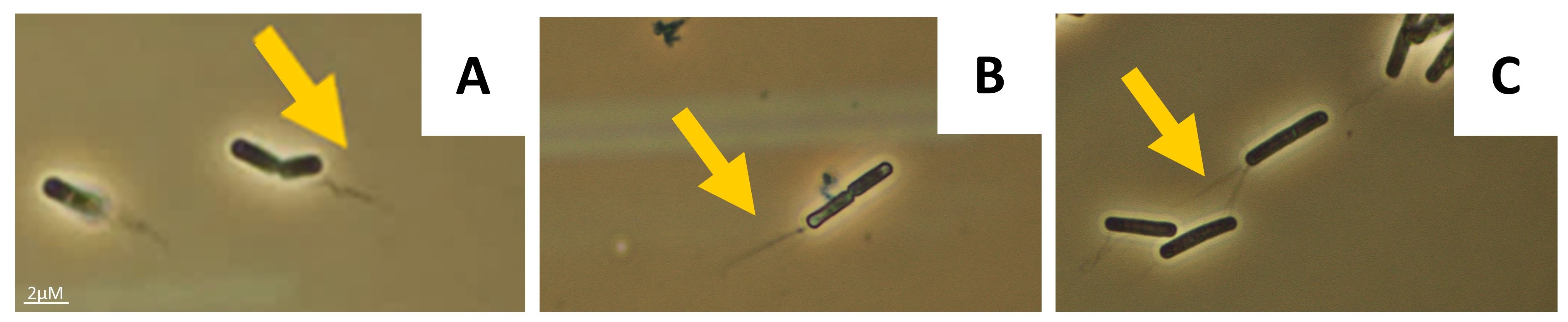

Supplement: Supplementary file 1 — Additional file 1: Supplementary Figure S1. Heat regulon genes/proteins in Bacillus cereus ATCC14579. Supplementary Figure S2. Flagella quick staining of wt cells (A), ΔsigB cells (B) and Δbc1009 cells (C). Flagella indicated with yellow arrow. Supplementary Figure S3. BC1009- dependent proteins after heat shock. Supplementary Table S1. Oligonucleotides used in this study. Supplementary Table S2A. Induced and downregulated proteins upon heat shock (30°C to 42°C) in Bacillus cereus ATCC14579 wt. Supplementary Table S2B. Induced and downregulated genes upon heat shock (30°C to 42°C) in Bacillus cereus ATCC14579 wt. Supplementary Table S3B. SigB-dependent induced and downregulated genes upon heat shock (30°C to 42°C) in Bacillus cereus wt cells versus ΔsigB and Δbc1009 mutants. Supplementary Table S4A. Bc1009-dependent proteins in Bacillus cereus upon heat shock (30°C to 42°C) in wt cells versus Δbc1009 mutant. Supplementary Table S4B. Bc1009-dependent genes in Bacillus cereus upon heat shock (30°C to 42°C) in wt cells versus Δbc1009 mutant. Supplementary Table S5A. Differentially regulated SigB- dependent or SigB and Bc1009-dependent proteins in non-heat-stressed condition at 30°C in B. cereus ATCC14579 wt cells versus ΔsigB and Δbc1009 mutants. Supplementary Table S5B. Differentially regulated SigB- dependent or SigB and Bc1009-dependent genes in non-heat-stressed condition at 30°C in B. cereus ATCC14579 wt cells versus ΔsigB and Δbc1009 mutants. [file 12866_2023_2783_MOESM1_ESM.zip › Figure S2 flagella staining pictures of wt and mutants revised.jpg]

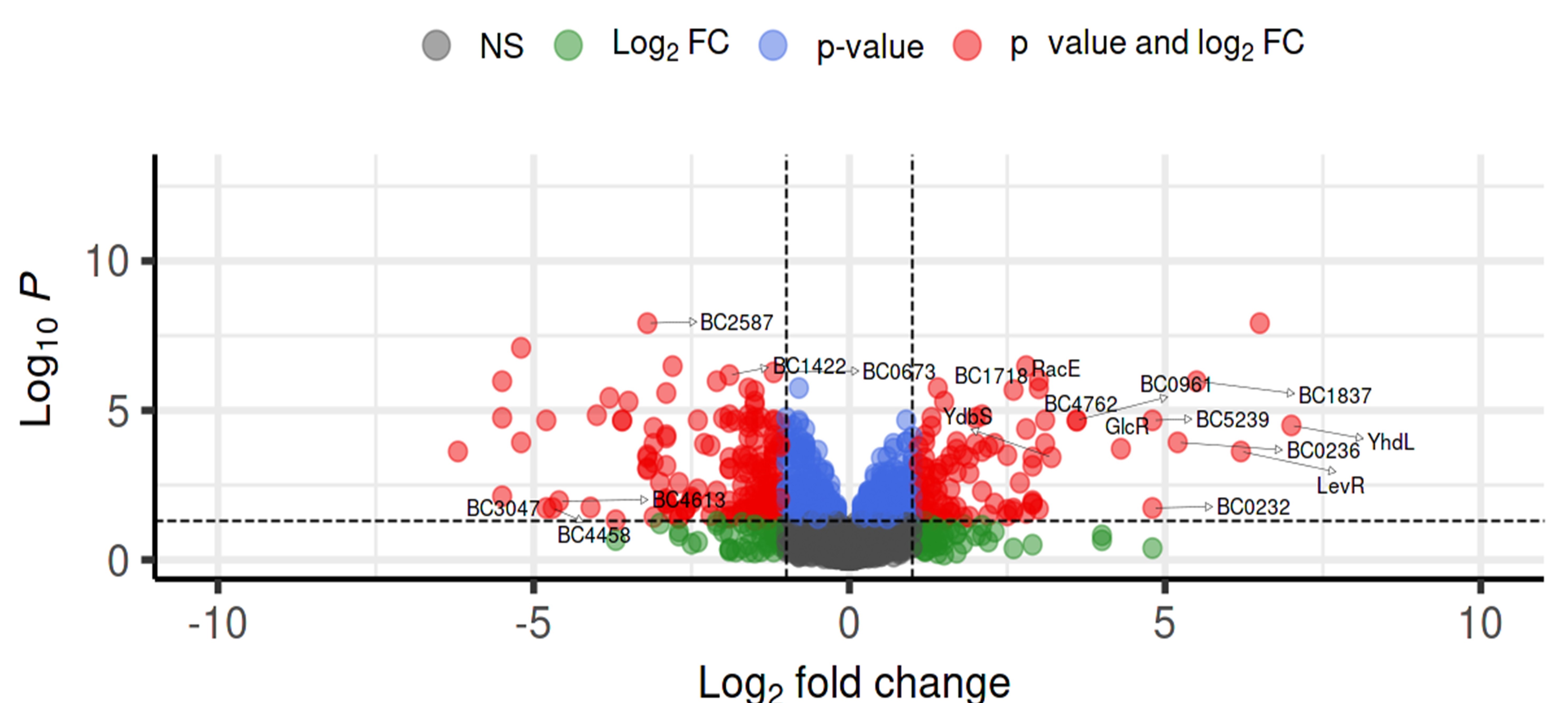

Supplement: Supplementary file 1 — Additional file 1: Supplementary Figure S1. Heat regulon genes/proteins in Bacillus cereus ATCC14579. Supplementary Figure S2. Flagella quick staining of wt cells (A), ΔsigB cells (B) and Δbc1009 cells (C). Flagella indicated with yellow arrow. Supplementary Figure S3. BC1009- dependent proteins after heat shock. Supplementary Table S1. Oligonucleotides used in this study. Supplementary Table S2A. Induced and downregulated proteins upon heat shock (30°C to 42°C) in Bacillus cereus ATCC14579 wt. Supplementary Table S2B. Induced and downregulated genes upon heat shock (30°C to 42°C) in Bacillus cereus ATCC14579 wt. Supplementary Table S3B. SigB-dependent induced and downregulated genes upon heat shock (30°C to 42°C) in Bacillus cereus wt cells versus ΔsigB and Δbc1009 mutants. Supplementary Table S4A. Bc1009-dependent proteins in Bacillus cereus upon heat shock (30°C to 42°C) in wt cells versus Δbc1009 mutant. Supplementary Table S4B. Bc1009-dependent genes in Bacillus cereus upon heat shock (30°C to 42°C) in wt cells versus Δbc1009 mutant. Supplementary Table S5A. Differentially regulated SigB- dependent or SigB and Bc1009-dependent proteins in non-heat-stressed condition at 30°C in B. cereus ATCC14579 wt cells versus ΔsigB and Δbc1009 mutants. Supplementary Table S5B. Differentially regulated SigB- dependent or SigB and Bc1009-dependent genes in non-heat-stressed condition at 30°C in B. cereus ATCC14579 wt cells versus ΔsigB and Δbc1009 mutants. [file 12866_2023_2783_MOESM1_ESM.zip › Figure S3- BC1009- dependent proteins after heat shock.jpg]
